# Driving Forces in the Formation of Paracetamol Cocrystals and Solvate with Naphthalene, Quinoline and Acridine

**DOI:** 10.3390/molecules29184437

**Published:** 2024-09-18

**Authors:** Tadeusz M. Muzioł, Emilia Bronikowska

**Affiliations:** Faculty of Chemistry, Nicolaus Copernicus University in Toruń, Gagarina 7, 87-100 Toruń, Poland; 307432@stud.umk.pl

**Keywords:** paracetamol, coformer, single crystal XRD, Hirshfeld surface, energy calculations, XAS for N and O K edges

## Abstract

Paracetamol is an important analgesic and antipyretic drug showing poor tabletability. Among the various approaches used to improve this property, understanding the forces that govern the crystal packing is revealed to be crucial. We prepared three stable compounds: (par)_2_∙(nap) (**1**), (par)∙(quin) (**2**), and (par)∙(acr) (**3**) (nap—naphthalene, quin—quinoline, acr—acridine) being cocrystals or solvate. The structural studies showed that all the reported compounds are composed of alternately arranged layers of paracetamol and coformer. Several supramolecular motifs in the paracetamol layer were identified: $R_{4}^{4}$(22) in (**1**); $R_{6}^{4}$(20) and $R_{2}^{2}$(8) in (**2**); and $R_{2}^{2}$(8), $R_{4}^{2}$(12), and $R_{4}^{4}$(26) rings in (**3**). The stability of the crystal network was studied by interactions analysis performed by Hirshfeld surface and fingerprint approaches and the energy between the closest units in the crystal network was calculated. It showed that the strongest interactions were found between blocks connected by N-H⋯O=C and O-H⋯O/N hydrogen bonds due to an important coulombic factor. The dispersive energy becomes important for tail-to-tail (and head-to-tail) arranged paracetamol units, and it prevails in the case of stacking interactions between coformer molecules. The importance of dispersive forces increases with the size of the aromatic system of the coformer. XAS studies confirmed the successful preparation of compounds and provided some details about electron structure.

## 1. Introduction

Paracetamol (acetaminophen, par) is an analgesic and antipyretic drug used for the treatment of headaches, muscle aches, arthritis, backache, and fevers [1,2,3] Its market exceeds one billion US dollars [4,5]. There are three known polymorphs of paracetamol: form I (monoclinic, *P*2_1_/*n*) is thermodynamically stable whereas form II (orthorhombic, *Pcab*) and form III (orthorhombic, *Pca*2_1_) are metastable [6]. For pharmaceutical applications, the stable form I is selected. Its structure consists of corrugated hydrogen-bonded layers resulting in poor tabletability because of its poor compression properties [4,7]. To overcome the problems related to poor solubility and tabletability, different approaches are proposed, especially for systems containing active pharmaceutical ingredients (APIs) [8,9,10]. The solubility can also be improved by the preparation of amorphous material or the conversion of crystalline one into the amorphous sample, but usually, the lower stability of amorphous forms is a significant drawback of this method [11,12]. Compression properties and tabletability of paracetamol under commercially applied pressures were enhanced by ultrasound-assisted crystallization, leading to micro- and nanocrystals [4]. An alternative approach involves crystal engineering enabling the design of multicomponent crystalline materials, namely cocrystals, solvates, and salts [4,6,8,12,13,14,15,16,17,18,19]. The difference in the pKa might be a useful criterion to predict if cocrystal or salt will be formed [7,20]. The former will be obtained if this difference is smaller than −1, and salts crystallize if ΔpK_a_ > 4. Salts can be obtained if dissociable groups are present in the components [21]. In the case of paracetamol, such easily ionizable groups are missing and, moreover, amide salts are susceptible to hydrolysis. Such salts can be prepared with strong acids and hydrochlorides are the most popular salts among APIs because of better bioavailability and a better dissolution rate. They easily form crystals due to enhanced electrostatic interactions and usually reveal better solubility [14]. Therefore, ca. 40% of drugs are administrated as salts. The discussion about the cocrystal term has lasted for a long time, resulting in the gradual broadening of its meaning [22,23]. Currently accepted by the IUCr, the definition states that a cocrystal is a “solid consisting of a crystalline single-phase material composed of two or more different molecular and/or ionic compounds generally in a stoichiometric ratio which are neither solvates nor simple salts” (Online Dictionary of Crystallography). Cocrystal preparation is aimed at improving physicochemical properties (solubility, melting point, dissolution rate, moisture uptake, stability, bioavailability, and tabletability), which usually differ significantly from their components [7,8]. Cocrystal preparation is challenging and phase diagrams can be very helpful because they enable the identification of stability region and crystallization conditions, a process consisting of two steps—nucleation and crystal growth [17,24]. It allows for the preparation of (par)_2_∙(pyr) solvate, which is stable only in a very narrow range of crystallization conditions. There are many methods used for cocrystal synthesis, e.g., solvent evaporation, flash-freezing, freeze-drying, anti-solvent addition, dry grinding, liquid-assisted grinding (LAG), thermal cocrystallization, rapid precipitation, spray-drying, electrospray crystallization, and microspacing in-air sublimation (MAS) [4,17,21,24,25,26,27]. Cocrystal formation requires careful selection of coformers based on their functional groups, molecular shape, and size, especially if ternary or quaternary cocrystals are designed [12,28,29]. The hierarchy of the synthons might be helpful in predicting the interaction pattern [30]. Synthon formation can be detected by different methods, mainly XRD but also by IR spectroscopy as it was reported for amid⋯acid arrangement [31]. Very recently, advanced theoretical considerations based on crystal structure prediction (CSP) were also applied. Those methods are able to take into account the crystallinity term in crystal formation since it is a long-range order object. Those crystals are maintained by numerous interactions, mainly hydrogen bonds and π-π (among them stackings) interactions but also dipole-dipole and halogen. In salts, electrostaticity is also important [8,21,32]. In the case of paracetamol, the cocrystal preparation aims to improve the structure of form I with paracetamol molecules connected by hydrogen bonds arranged in very folded layers [4]. It was shown that flat layers improve compression properties and tabletability [6,14,18,21]. However, the process is not completely understood and the layer smoothness is not the only factor affecting tabletability because, for (par)∙(TMG) (trimethylglycine) and (par)∙(TOSA) (*p*-toluenesulfonic acid), the structures do not reveal flat layers but compression was superior to form I paracetamol [6,7]. 

There are many known cocrystals and salts of paracetamol with different coformers. Those generally recognized as safe (GRAS) are important for pharmaceutical applications as well as other coformers to investigate intermolecular interactions, synthons, and packing patterns [2,6,7,8,11,12,13,16,19,21,24,26,33,34,35,36]. In the current paper, we present three structures of compounds given by the formula (par)_2_∙(nap) (**1**), (par)∙(quin) (**2**), and (par)∙(acr) (**3**) (nap—naphthalene, quin—quinoline, acr—acridine). Due to the toxicity of the selected planar coformers, our main aim was to prepare monocrystals, determine structures, study intermolecular interactions, form patterns, and to get a deeper understanding of the crystal packing—an important issue in terms of rational cocrystal design. A detailed analysis of intermolecular interactions being a driving force responsible for the crystal network stability is provided. It is supported by Hirshfeld surface analysis and energy calculations for interacting moieties. All those compounds were characterized by IR spectroscopy and thermal analysis. Finally, the electronic structure of those cocrystals was probed by XAS. 

## 2. Results and Discussion

### 2.1. Cocrystal Preparation and Thermal Analysis

The preparation of cocrystals was attempted by using three par:coformer stoichiometric ratios (2:1, 1:1, 1:2) and the desired products were obtained in only one case: 2:1 for (**1**), and 1:1 for (**2**) and (**3**). We also performed trials to get crystals in paracetamol-isoquinoline and paracetamol-anthracene systems but all attempts failed and we crystallized only a mixture of starting materials. 

Thermal analysis for (**2**) and (**3**) showed complete decomposition of the cocrystals (the mass residue is close to 0) (Figure S1). Those processes are completed at ca. 330 °C with the mass residue being at ca. 2% and further heating results in dropping to a value below 0.2%. The decomposition process is rather complicated and, at least for (**2**), we observe two steps. The first one is completed at ca. 200 °C and it is related to the loss of quinoline molecules (experimental: 46.15%, theoretical: 46.07%). In the second step, paracetamol decomposes. For (**3**), we cannot identify unequivocally two steps but TG and DTA curves show that this process could also proceed in two steps with the paracetamol decomposition following immediately after the first step (acridine release). Those decomposition patterns suggest satisfactory stability of the obtained cocrystals for (**2**) and (**3**), which can be related to the formed interactions in the crystal network (see Section 2.3). For (par)_2_∙(nap), DSC measurements performed by Karki et al. [21] showed two peaks corresponding to naphthalene release at ca. 130 °C and monoclinic paracetamol formation. 

### 2.2. IR Spectroscopy

For all three obtained compounds, the bands of paracetamol are visible in similar ranges, with possible slight shifts due to the robust hydrogen bond networks (Figure S2). For paracetamol, the stretching bands originating from the methyl group are observed in the ranges of 3319–3328 cm^−1^ and 3260 cm^−1^. In the range of 1608–1610 cm^−1^, stretching vibrations from C=C are observed and from C-O at 1643–1655 cm^−1^ [2,6,7,11,34,37]. Bending vibrations originating from C-H are visible for all structures around 1504 cm^−1^, while those from C-C are in the range of 1437–1448 cm^−1^. Stretching vibrations originating from C-H are visible at approximately 1369–1376 cm^−1^, while those from C-N are visible at 1230–1252 cm^−1^ and 952–976 cm^−1^. Aromatic ring vibrations (in-plane and out-of-plane) are observed at around 828 and 508–511 cm^−1^. For (**2**), the spectrum was compared with the one obtained by Karki et al. [21] showing bands at the same values. 

For naphthalene, quinoline, and acridine, bending vibrations originating from hydrogens that are out-of-plane are observed in the range of 1109–1115 cm^−1^ [37,38,39,40,41,42]. Vibrations originating from C-C and C-H in their aromatic rings are visible in the ranges of 787–795 cm^−1^ and 589–596 cm^−1^. Stretching vibrations originating from the C-N of quinoline and acridine are observed at 1306–1314 cm^−1^.

### 2.3. Structural Studies

#### 2.3.1. Structure of (par)_2_∙(nap) (**1**)

**Figure 1:**
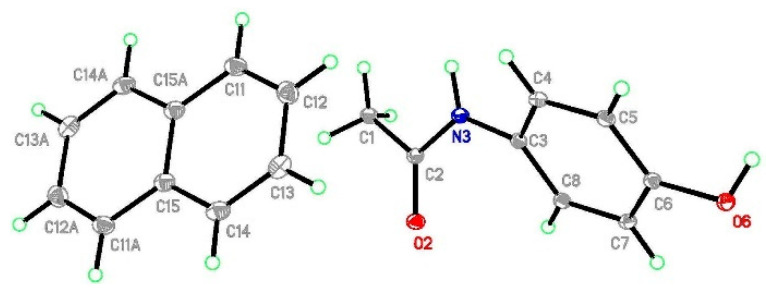
The structure of (par)_2_∙(nap) (**1**) with ellipsoids at the level of 30% probability.

**Figure 2:**
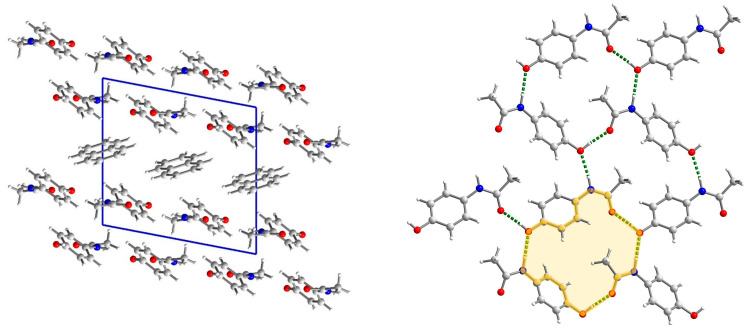
The crystal network of (par)_2_∙(nap) (**1**) along the *b* axis (**left**). The structure of the paracetamol layer with, marked in green, O-H_par_⋯O=C_par_ and N-H_par_⋯O-H_par_ hydrogen bonds involved in $R_{4}^{4}$(22) ring formation (**right**). The $R_{4}^{4}$(22) ring is marked in yellow.

**Figure 3:**
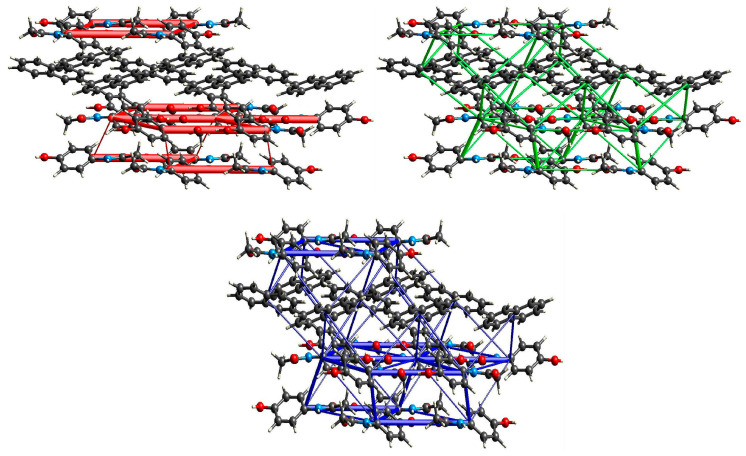
Energy of interactions between molecules in the crystal network of (par)_2_∙(nap) (**1**) calculated in CrystalExplorer [47] (red—electrostatic energy, green—dispersive energy, and blue—total energy).

This structure was briefly described by Karki et al. mainly in terms of layer structure, which is important for tabletability properties [21]. Therefore, we will provide our description supplemented by energy network calculations. (par)_2_∙(nap) (**1**) cocrystals crystallize in a monoclinic *P*2_1_/*c* space group with all atoms in general positions (Figure 1). In the asymmetric unit, there is a single paracetamol and half of a naphthalene molecule. In packing along the *b* axis, the naphthalene layer separates the double layers of paracetamol (Figure 2 and Figure S3). Paracetamol molecules form a robust network of hydrogen bonds. Every molecule forms four hydrogen bonds with four adjacent paracetamol molecules. They are created via the hydroxyl group acting as the donor and acceptor of the hydrogen bond, and the amide group also acts as a donor (−NH) and acceptor (C=O) of the hydrogen bond (Table S1). This pattern shows $R_{4}^{4}(22)$ rings created by four paracetamol molecules [43] (Figure 2). Interactions between paracetamol layers are assured by C8-H8a⋯π[−x, 1−y, 1−z] interactions created by the phenyl ring and reinforced by other weak contacts between head-to-tail oriented molecules (Figure S3, Table S2). More complete information about intermolecular interactions is provided by Hirshfeld surface analysis showing that, in the case of both paracetamol and naphthalene, H⋯H and C⋯H contacts are the most numerous (Figure S4), highlighting that dispersive forces should be crucial [44,45,46]. The most comprehensive knowledge about interaction strength is given by energy calculations between adjacent blocks [47]. They clearly indicate that both factors—electrostatic and dispersive forces—are crucial in crystal network formation (Figure 3). Three pairs of molecules provide the main contribution to the energy of the crystal network. In two of them, the predominant role is played by O-H_par_⋯O=C_par_ and N-H_par_⋯O-H_par_ hydrogen bonds found in the paracetamol layer. This is confirmed by a Hirshfeld surface analysis, which shows many H⋯O interactions between adjacent paracetamol molecules and spikes on the fingerprint corresponding to the above-mentioned short and well-oriented hydrogen bonds (Figure S4). Those interactions are manifested also by red spots on the Hirshfeld surface of the paracetamol molecule. For the head-to-tail oriented paracetamol molecules coming from adjacent layers, the van der Waals forces prevail (Figure S4). C-H⋯O interactions are much less numerous between naphthalene and paracetamol molecules, indicating that dispersive forces are crucial for naphthalene. The aromatic systems present in both components of the cocrystal highlight the π-π interactions between strongly inclined C3 phenyl and C11 naphthalene rings (Table S3). The interaction pattern is completed by C2-H2⋯naphthalene ring, C8-H8⋯phenyl ring, and C14-H14⋯phenyl ring interactions. Nevertheless, the energy of par⋯nap interactions is much lower due to the small impact of the electrostatic factor. In the naphthalene layer, the separation between nap molecules is significant (the shortest distance between centroids is 7.228 Å) and there is no evidence of any important interactions between nap molecules. Hence, the interactions between them are negligible. This weakness of interactions between naphthalene molecules as well as between naphthalene and paracetamol molecules can explain the observation made by Karki et al. that naphthalene is gradually released from the crystal network when the product is heated from 30 to 180 °C, resulting in conversion to the monoclinic form I [21].

#### 2.3.2. Structure of (par)∙(quin) (**2**)

**Figure 4:**
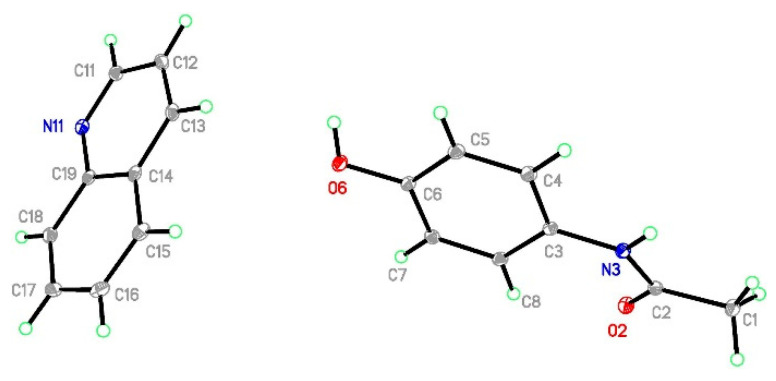
The structure of (par)∙(quin) (**2**) with ellipsoids at the level of 30% probability.

**Figure 5:**
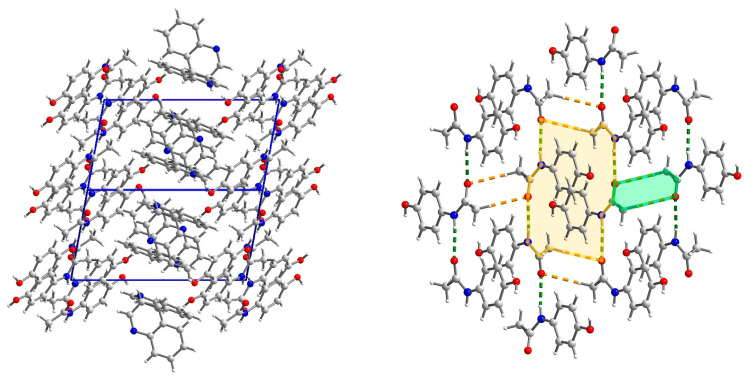
The crystal network of (par)∙(quin) (**2**) along the [011] direction shows an alternate arrangement of paracetamol and quinoline layers (**left**). Interaction in paracetamol layers (**right**). In the par layer, the hydrogen bonds are marked in green and C-H⋯O in orange. Two rings ($R_{6}^{4}$(20) (yellow) and $R_{2}^{2}$(8) (green)) are clearly visible.

**Figure 6:**
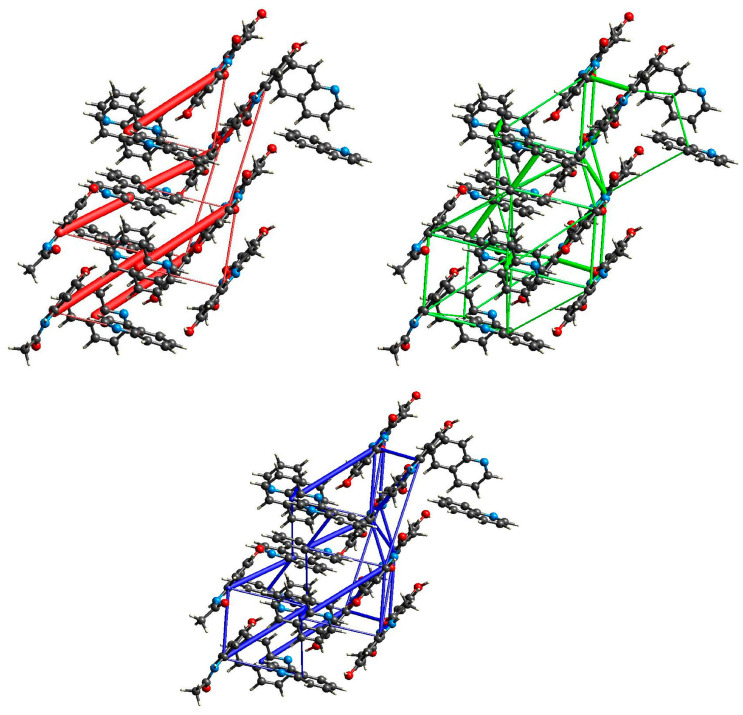
Energy of interactions between molecules in the crystal network of (par)∙(quin) (**2**) calculated in CrystalExplorer [47] (red—electrostatic energy, green—dispersive energy, and blue—total energy).

(par)∙(quin) (**2**) solvate crystallizes in a monoclinic *P*2_1_/*c* space group with all atoms in the general position (Figure 4). In the asymmetric unit, there is one paracetamol and one quinoline molecule. In packing along the [011] direction, an alternate arrangement of paracetamol and quinoline layers is observed (Figure 5). The paracetamol (10$\bar{1}$) layer is composed of chains running along the *b* axis and formed by the N-H_par_⋯C=O_par_ hydrogen bond (N3-H3⋯O2[2−x, ½+y, −½−z]) (Figure S5, Table S4) [24]. To enable the formation of such contact, two molecules are found in head-to-tail orientation and their phenyl rings form a dihedral angle of 6.7°, whereas the O6-N3-N3[2−x, ½+y, −½−z]-O6[2−x, ½+y, −½−z] torsion angle is −179.6° (Figure S5, Table S5). Subsequently, the layer is created via C1-H1C⋯O2[2−x, −y, −1−z] hydrogen bonds. It results in new topological features, namely big ($R_{6}^{4}$(20)) and small ($R_{2}^{2}$(8)) rings (Figure 5). The smaller one is created by the CH_3_-CO(NH) tails of two par molecules, whereas the bigger one requires six molecules connected by four strong and two weak hydrogen bonds. This layer is reinforced by stacking interactions between phenyl rings and by C2-O2⋯C3 phenyl ring contacts (Table S6). The coformer layer is formed from pairs of quinoline molecules related by the inversion center involved in strong stacking interactions (Table S5). Subsequently, the dimer is surrounded by four heavily inclined dimers (71.53°) and forming π-π interactions. Hence, the stacking cannot propagate due to those capping molecules. The paracetamol and quinoline layers are cross-linked by an O6-H6_par_⋯N11_quin_[1−x, ½+y, ½−z] hydrogen bond, a C11-H11_quin_⋯C3_par_[1−x, −y, −z] phenyl ring, and π-π interactions between strongly inclined (67.60°) phenyl and quinoline rings. The whole landscape of interactions in the crystal network is dominated by H⋯H as well as H⋯C contacts for both paracetamol and quinoline molecules (Figure S6). However, due to the presence of quite numerous H⋯O interactions and spikes occurring on a fingerprint indicating some short contacts related to strong hydrogen bonds, we can expect that the electrostatic factor will be important. Another spike is observed for H⋯N interactions, which is also manifested as red spots on Hirshfeld surfaces. Similarly, such a feature also occurs for quinoline fingerprints and it is also related to hydrogen bonds. In paracetamol, the amide group is a donor and an acceptor and the hydroxyl group acts solely as a donor. The interaction energy is the highest for molecules involved in hydrogen bonding, namely paracetamol and quinoline connected via the O-H_par_⋯N_quin_ bond and two paracetamol molecules joined by the N-H_par_⋯O=C_par_ bond due to the significantly higher impact of coulomb energy (Figure 6). Hence, the substitution of carbon with nitrogen significantly improved the interaction energy between layers formed in (**2**). Nevertheless, dispersive forces also play an important role because the energy of π-π interactions between two quinoline molecules is still high.

#### 2.3.3. Structure of (par)∙(acr) (**3**)

**Figure 7:**
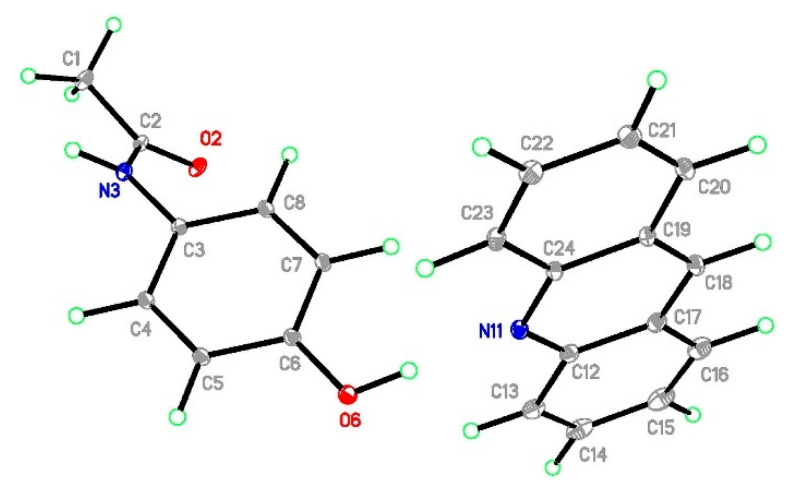
The structure of (par)∙(acr) (**3**) with ellipsoids at the level of 30% probability.

**Figure 8:**
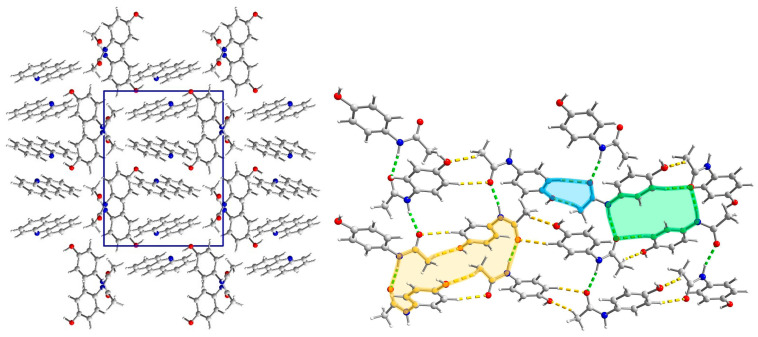
The crystal network of (par)∙(acr) (**3**) along the *c* axis shows alternately arranged paracetamol and acridine layers (**left**). The paracetamol layer of (**3**) is composed of chains (molecules connected by hydrogen bonds in green) which are crosslinked by C-H⋯O hydrogen bonds (in orange) (**right**). New supramolecular motifs ($R_{2}^{2}$(8) rings in blue, $R_{4}^{2}$(12) in green, and $R_{4}^{4}$(26) in yellow) were formed.

**Figure 9:**
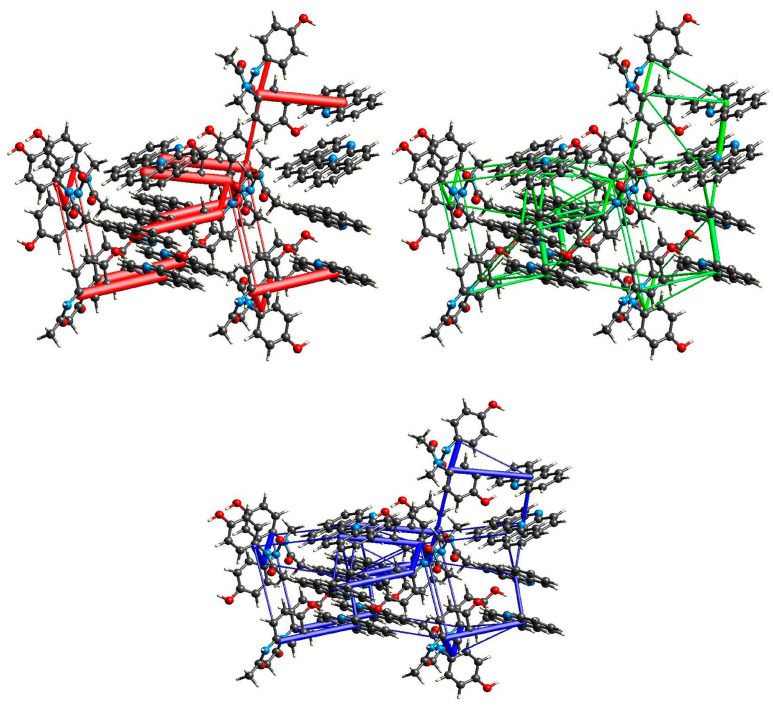
Energy of interactions between molecules in the crystal network of (par)∙(acr) (**3**) calculated in CrystalExplorer [47] (red—electrostatic energy, green—dispersive energy, and blue—total energy).

(par)∙(acr) (**3**) crystallizes in a monoclinic *P*2_1_/*c* space group with all atoms in general positions and one paracetamol and one acridine molecule in the asymmetric unit (Figure 7). In packing along the *c* axis, we observe alternately arranged acridine and paracetamol layers (Figure 8). Paracetamol molecules form chains via N-H_par_⋯O_par_ hydrogen bonds formed between amide groups (Figure 8 and Figure S7). The molecules in the chain show coplanar phenyl rings but the paracetamol axes given by O6→N3 vectors are strongly tilted (the dihedral O6-N3-N3[x, ^3^/_2_−y, −½+z]-O6[x, ^3^/_2_−y, −½+z] angle is −96.65°). Those chains are crosslinked into a layer by weak C1-H1C⋯O6[2−x, ½+y, ½−z] and C5-H5⋯O2[2−x, −½+y, ½−z] hydrogen bonds (Table S7). As a consequence, new structural features emerge, namely three rings: $R_{2}^{2}$(8), $R_{4}^{2}$(12), and $R_{4}^{4}$(26). The smallest one was identified relatively often in many structures, e.g., in carboxylic acids [43]. In (**2**), it is composed of two tail-to-tail oriented paracetamol molecules to enable the formation of two weak C-H_par_⋯O_par_ hydrogen bonds. Two bigger motifs were not observed in the reported structures. They occur due to different mutual paracetamol orientations in (**2**) and (**3**) as indicated by the O6-N3-N3-O6 torsion angle (−179.6 and −96.65°, respectively). $R_{4}^{2}$(12) is composed of four molecules connected by two strong and two weak hydrogen bonds, whereas in $R_{4}^{4}$(26) formation, four par molecules are also involved and connected by six hydrogen bonds (two strong and four weak). Similarly to (**2**), O6 oxygen atoms act solely as hydrogen bond donors. Hence, the paracetamol OH group is not involved in the formation of the par layer, but it forms very short and well-oriented hydrogen bonds with acridine molecules. Moreover, there are also C21-H21⋯O2[1−x, 1−y, −z] and C22-H22⋯O6[−1+x, ^3^/_2_−y, −½+z] hydrogen bonds between the acr and par molecules. Hence, acridine creates hydrogen bonds with two adjacent paracetamol layers. The par⋯acr interaction pattern is completed by perpendicularly oriented rings of both molecules and also by C14-H14⋯C3[x, y, 1+z] phenyl ring contact (Tables S8 and S9). Therefore, interactions between both layers are much stronger than in (**1**). In the acridine layer, the molecules interact via stacking interaction as well as π-π interactions between perpendicularly oriented acr moieties. Hence, stacking orientation is observed only for a pair of molecules and this motif does not propagate. It highlights that this layer is much more robust than nap in (**1**). The Hirshfeld surface showed that, in (**3**), for both API and coformer, the vast majority of contacts are created between two hydrogen atoms and hydrogen and carbon atoms (Figure S8). For C⋯H in acridine, there is also a pale red spot indicating that some short contacts are observed. Nevertheless, especially for paracetamol, H⋯O contacts are also numerous and there are many red spots corresponding to hydrogen bonds observed on fingerprints as spikes. H⋯N contacts are not so often encountered, but they are also important as they correspond to hydrogen bonds (red spots and spikes on the corresponding figures). The energy calculations showed the principal interactions related to the most important features observed in the reported structure (Figure 9). The strongest interactions correspond to par⋯acr pair connected by O6-H6_par_⋯N11_acr_ hydrogen bonds. Slightly lower energy is found for two paracetamol molecules connected by the N3-H3⋯O2[x, ^3^/_2_−y, −½+z] hydrogen bond. In both cases, the most significant is the electrostatic factor related to hydrogen bonding, whereas the dispersive term is much lower. Contrary, for stacking interactions between two well-oriented acridine molecules, the van der Waals forces are crucial whereas the electrostatic energy is negligible. Interactions between two acridine molecules oriented perpendicularly are much lower but still important. It shows also that the interaction energy depends on the mutual orientation of two moieties.

#### 2.3.4. Structural Studies of Selected Coformers

Apart from (**1**)–(**3**), two other compounds—(par)∙(phe)_2_ (phe—phenazine) [21] and (par)_2_∙(pyr) [pyr—pyridine) [24]—are known. All those compounds show significantly different crystal networks despite coformers being closely related and quin from nap as well as phe from acr differs only in one atom due to C→N substitution, proving that even such seemingly small changes substantially affect the obtained topologies. The stoichiometric ratio par:phe being 1:2 indicates that the paracetamol molecules should be well separated in the crystal network. This is confirmed by the crystal packing with paracetamol located in the voids between the phenazine columns (Figure S9). In (par)∙(phe)_2_, hydrogen bonds enabled cocrystal formation with a Z-shaped arrangement of molecules [21]. The most important interactions are created by paracetamol and phenazine molecules joined by O-H_par_⋯N_phe_ and N-H_par_⋯N_phe_ hydrogen bonds, whereas C=O_par_ and H-O_par_ groups act as acceptors of hydrogen atoms from the C-H_phe_ groups. Interactions between large aromatic systems forming columns are also efficient due to the dispersive forces. The significant separation between paracetamol molecules results in usually weak par⋯par interactions. However, for two par molecules oriented in the tail-to-tail mode, the interactions are quite strong due to the synergetic effect of the dispersive and electrostatic forces (Figure S10).

Pyridine is the simplest compound among nitrogen heterocycles. Its (par)_2_∙(pyr) solvate was reported with pyridine molecules hidden in the cavity of the crystal network due to the significantly smaller size of this molecule [24]. Chains composed of paracetamol molecules are created by N-H_par_⋯O=C_par_ hydrogen bonds and OH groups acting as both donors and acceptors of hydrogen bonds. Those features are similar to the hydrogen bonds observed in (**2**) and (**3**), and the hydroxyl group creates interactions with both components—paracetamol and coformer. However, the pyridine solvate hydroxyl group is involved in strong O-H_par_⋯O-H_par_ hydrogen bonds, whereas in (**3**), one of those bonds is weak and involves the C-H group. Despite the small size of pyridine in (par)_2_∙(pyr) solvate, both factors—electrostatic and dispersive—are very important. The former one is decisive in O-H_par_⋯N_pyr_ hydrogen bond formation (with an energy similar to paracetamol molecules involved in the bond formation), whereas the latter prevails in pyr⋯pyr stacking interactions.

We defined some molecular arrangements observed in the reported structures and also in the cited structures [21,24], among them molecules related by stacking interactions, N-H⋯O=C hydrogen bonds, and tail-to-tail or head-to-tail oriented molecules (Tables S10 and S11). It can be seen that paracetamol usually forms at least three strong hydrogen bonds with the amide group acting as both donor and acceptor, whereas the hydroxyl group usually is a donor in hydrogen bonds. There are two common motifs, namely the N-H_par_⋯O=C_par_ hydrogen bond and interactions with a coformer possessing nitrogen atoms (O-H_par_⋯N_coformer_). In both those cases, the electrostatic factor related to hydrogen bonding is very important. Dispersive forces are important for molecules oriented in tail-to-tail (and head-to-tail) mode, especially in the case of stacking interactions. In the latter case, van der Waals forces prevail and their strength is dependent on both size (the smallest pyridine forms moderate strength interactions) and orientation (in naphthalene, due to significant separation and an unfavorable mutual position, the energy is negligible). However, a significant increase is observed between pyridine and quinoline, whereas for acridine and phenazine possessing additional rings, only a small further augmentation is noticed. We can see also that N-H⋯O and O-H⋯O hydrogen bonds, commonly called strong or classical hydrogen bonds, assure much more efficient interactions than C-H⋯O bonding. This analysis shows also that the observed supramolecular motifs consist of the moieties creating efficient interactions.

The conformation of the free paracetamol is expected to be flat [24]. However, it is expected to be altered due to crystal network influence. In the Cambridge Structural Database (version 5.45, updates June 2024), there are 113 entries with par molecules and 12 with parH^+^ cation (Figures S13 and S14). The C1-C2-N3-C3 dihedral angle usually adopts a value close to 180°, whereas the torsion angle changes in quite a broad range from 0.3–70.8° and 1.0–47.8° for par and parH^+^, respectively. Hence, the crystal network can substantially distort the conformation. Those values usually do not exceed 54° (there is only one outlier above 70° related to a strongly biased model obtained as a result of single-phase Rietveld refinement) [48]. In (**2**) (18.6°), it falls into the commonly detected range of values, whereas such bigger distortions, as in (**1**) (38.4°) and especially in (**3**) (47.8°), are relatively rare. For pyridine (1.0°) and phenazine (4.9°), these values are very small—in those cases, the packing does not distort the paracetamol conformation. It shows that paracetamol can be a versatile block capable of forming different topologies.

### 2.4. Powder Diffraction Experiments

**Figure 10:**
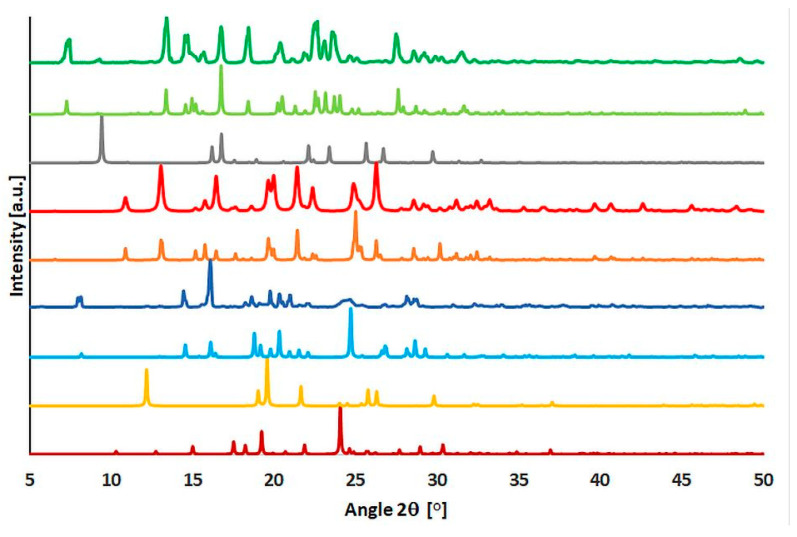
Powder patterns of (from bottom to the top): monoclinic paracetamol (brown), naphthalene (yellow), calculated (par)_2_∙(nap) (blue), experimental (par)_2_∙(nap) (dark blue), calculated (par)∙(quin) (light brown), experimental (par)∙(quin) (red), acridine (grey), calculated (par)∙(acr) (light green), and experimental (par)∙(acr) (green).

The powder experiments were performed to verify the purity of the obtained compounds and to verify the stability of those compounds. We have not noticed any significant changes after a few weeks of storage of those compounds. Diffraction patterns match well with those calculated using the final models of the reported compounds Figure 10). It should be noted a significant decrease in intensities above 35°. Moreover, we cannot see significant impurities coming from substrate molecules—paracetamol, naphthalene, and acridine. This proves that (**1**)–(**3**) are pure (par)_2_∙(nap), (par)∙(quin), and (par)∙(acr). The subtle changes in intensity or in peak positions might also be related to the difference in temperatures—powder experiments were performed at room temperature, whereas single crystal measurements were at 100 K.

### 2.5. XAS Results

**Figure 11:**
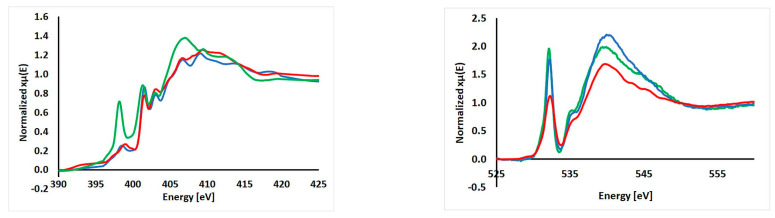
Normalized XANES spectra of nitrogen (**left**) and oxygen (**right**) K-edge for powdered samples of (**1**) (red), (**2**) (blue), and (**3**) (green) collected with TEY detector.

XAS spectra for the nitrogen K-edge were recorded in total electron yield (TEY) and total fluorescence yield (TFY) modes. Both detectors differ in the depth of sample probing—it is ca. 10 nm and 100 nm, respectively [49]. However, it should be noted that, in both cases, the spectra usually do not differ substantially, and only results for TEY mode will be presented and discussed. All spectra are rather complex, and there are at least five features in the range 398–415 eV, which are usually discussed (Figure 11) [50,51,52]. For (**1**) and (**2**), peak positions are similar, with one peak slightly below 400 eV and the main peak at 401.5 eV, which is followed by another one at 403.1 eV. Above 403 eV, a broad band starts with two peaks at 406.6 and 409.2 eV for naphthalene and 409.4 eV for quinoline cocrystals (Table S12). For (para)∙(acr) (**3**), those peaks are observed at 398.2, 401.3, 403.0, 407.0, and 409.4 eV. Hence, peak positions are quite similar, but this spectrum differs substantially in terms of the intensity ratio of the two first peaks. In (**3**), the peak at 398.2 eV is much more pronounced. For organic compounds, peaks below 405 eV are ascribed to 1s→π* transitions, and features above 405 eV correspond to 1s→σ* transitions. It should be noted that peaks located at lower energies are usually sharper [53]. The authors performed an excellent study of many reference compounds with different functional groups containing nitrogen. In the reported (**1**)–(**3**) compounds, the amide group is present in paracetamol and nitrogen atoms in a six-membered heterocyclic ring. According to the authors, for the amide group, the signals should occur below 400 eV and the main feature should be visible at 401.4 eV. This effect is due to C=O electrons dispersed over nitrogen atoms. In the case of aromatic heterocyclic rings, the resonance is expected at ca. 398.7 eV. It agrees well with our observations. We showed that additional nitrogen atoms located in aromatic rings significantly affect the spectrum, and hence, the successful formation of cocrystals can also be confirmed by XAS.

For the O K-edge, the spectra collected using TEY and TFY modes are similar. Hence, only those with TEY detectors will be discussed in detail. For all cocrystal XAS spectra for oxygen, the K-edge showed three features (Table S13). The main peak occurred at ca. 532.2 eV followed by a feature at ca. 535.3 eV and a broad band with a maximum at ca. 539.9 (Figure 11). Those spectra are very similar in terms of peak positions, intensities, and profiles because only paracetamol contains two oxygen atoms—one in the hydroxyl group and another one in the amide group. The presence of both groups produces specific peaks and enables their identification [54]. The carbonyl group gives a signal at lower energies than in the case of a hydroxyl group. They are separated by 2–3 eV and this value fits the difference observed in our spectra. Hence, we can identify both functional groups and confirm the presence of paracetamol in cocrystals.

## 3. Materials and Methods

### 3.1. Materials and General Procedure

All reagents used in the cocrystal preparation were of analytical grade and used without further purification. Elemental analysis (C, H, N) was carried out with a Vario MACRO analyzer. The IR spectrum was recorded on an FT-IR Vertex 70V (Bruker, MA, USA) spectrophotometer working in the ATR mode in the 4000–400 cm^−1^ region. Derivatograms were collected on an SDT 2600 TA Instruments by simultaneous thermogravimetric analysis (TGA) and differential thermal analysis (DTA) in a stream of air up to 1000 °C. The powder diffraction patterns were collected using a Phillips X’Pert Pro diffractometer equipped with an X’Celerator Scientific RTMS detector using CuK_α_ radiation. The diffractograms were registered with a step of 0.017° 2θ and an exposure time of 75 s in the range 5 < 2θ < 50°. The theoretical spectra were calculated in Mercury [55] in the range 5–50° 2θ and assuming the default FWHM equals 0.1° for (**1**)–(**3**). X-ray absorption spectra were recorded at the National Synchrotron Radiation Centre SOLARIS at the bending magnet PIRX beamline for N (380–440 eV) and O (515–580 eV) K edges. The powder samples were finely pulverized and attached to double-sided adhesive conductive graphite tape. The measurements were carried out with the step size of 0.2 eV for the pre-edge region, 0.1 eV for the edge regions, and 0.5 eV for the high-energy part. The data sets were collected at room temperature in an ultra-high vacuum (UHV) using total electron yield (TEY) and total fluorescence yield (TFY) modes. The measurements were repeated at least threefold. The data were processed using the ATHENA program from the Demeter package [56]. The intermolecular interactions analysis was performed in CrystalExplorer 21.5 using the Hirshfeld surface and fingerprints [44,45,46]. The interaction energy between adjacent blocks was also calculated in CrystalExplorer 21.5 using B3LYP functional and 6-31G(d,p) basis set [47,57,58,59].

### 3.2. Preparation of (par)_2_∙(nap)

In total, 0.1927 g (1.5 mmol) of naphthalene and 0.4536 g (3 mmol) of paracetamol were weighed and then dissolved in 20 mL of a mixture of chloroform and methanol (1:1 ratio). The solution was stirred for 20 min, then left at room temperature to evaporate slowly the solvent. Colorless crystals, suitable for diffraction experiments, emerged after five to six days. The synthetic procedure was repeated also in par:nap stoichiometric ratios of 1:2 and 1:1 but the product was not obtained. Found C: 72.49; H: 6.09; N: 6.10%. Calc. for (C_8_H_9_NO_2_)_2_(C_10_H_8_) C: 72.54; H: 6.09; N: 6.51%.

### 3.3. Preparation of (par)∙(quin)

In total, 1.95 mL (1.5 mmol) of quinoline and 0.4535 g (3 mmol) of paracetamol were measured, which were then dissolved in a 10 mL mixture of chloroform and methanol (1:1 ratio). The solution was stirred for 20 min, then left at room temperature to evaporate slowly. Yellowish crystals suitable for diffraction experiments emerged after five months. The synthetic procedure was repeated also in par:quin stoichiometric ratios of 2:1 and 1:2 but the product was not obtained. Found C: 72.45; H: 5.72; N: 9.65%. Calc. for (C_8_H_9_NO_2_)(C_9_H_7_N) C: 72.84; H: 5.75; N: 9.99%.

### 3.4. Preparation of (par)∙(acr)

In total, 0.5378 g (3 mmol) of acridine and 0.4532 g (3 mmol) of paracetamol were weighed and then dissolved in a 20 mL mixture of chloroform and methanol (1:1 ratio). The solution was stirred for 20 min, then left at room temperature to evaporate slowly. Colorless crystals, suitable for diffraction experiments, emerged after five to six days. The synthetic procedure was repeated also in par:acr stoichiometric ratios of 2:1 and 1:2 but the product was not obtained. Found C: 76.35; H: 5.48; N: 8.40%. Calc. for (C_8_H_9_NO_2_)(C_13_H_9_N) C: 76.34; H: 5.49; N: 8.48%.

### 3.5. Single Crystal X-ray Diffraction Measurements

The diffraction data for all compounds were collected at 100 K on a Rigaku XtaLAB Synergy (Dualflex) diffractometer with a HyPix detector. For (**1**) and (**2**), a monochromated CuKα X-ray source (λ = 1.54184 Å) was used; whereas, for (**3**), we observed fairly better diffraction, and hence, we decided to perform data collection using MoKα X-ray radiation (λ = 0.71073 Å) enabling high-resolution experiments. However, the data processing showed that useful diffraction reached only 0.7 Å resolution. Therefore, all three data sets were treated in the same manner. The data processing and the absorption correction were performed using CrysAlisPro [60]. The direct methods were used and the refinement was carried out with anisotropic displacement parameters [61]. Hydrogen atoms were located at calculated positions and refined with isotropic thermal displacement parameters fixed to a value 20 or 50% higher than the corresponding carbon atoms [62]. For (**2**), the extinction parameter was also refined. All figures were prepared in ORTEP [63], Mercury [55], and DIAMOND [64]. The data collection and refinement results have been summarized in Table S1, and selected bond lengths and angles are presented in Table S14. CCDC 2377795, 2377796, and 2377797 contain supplementary crystallographic data for (**1**), (**2**), and (**3**), respectively. These data can be obtained free from The Cambridge Crystallographic Data Centre via www.ccdc.cam.ac.uk/structures.

## 4. Conclusions

We prepared three compounds: (par)_2_∙(nap) (**1**), (par)∙(quin) (**2**) and (par)∙(acr) (**3**). These are cocrystals or solvates with paracetamol which is an important and commonly used antipyretic and analgesic drug. Elemental analysis, IR spectroscopy, PXRD, and thermal analysis revealed that those compounds are pure and stable. They undergo decomposition with the release of coformer molecules followed by paracetamol decomposition. XAS studies showed that this very modern method can be used to verify if cocrystals were successfully prepared, which also provides insight into their electron structure. Moreover, Lanzilotto et al. showed that it can be also used for tracing hydrogen bonding if the electronic states of two nitrogen groups are modified in different manners upon non-covalent interactions [65]. We presume that distinct hydrogen bonding can be responsible for the differences in the O K edge. The structural studies showed that all the reported compounds are composed of alternately arranged layers of paracetamol and coformer. The stability of the crystal network for (**1**)–(**3**), (par)_2_∙(pyr), and (par)∙(pyr)_2_ was studied by interactions analysis performed by the Hirshfeld surface and fingerprints approach and the energy calculations between the closest units in the crystal network.

Five different crystal packings were analyzed depending on the stoichiometry of the cocrystal. For the par:coformer ratio of 1:1, alternately arranged layers were usually found; whereas, for 2:1, the products depend on the coformer size—small pyridine was located in the network cavity, but for (**2**), layers were also observed. Finally, for 1:2 product—(par)∙(phe)_2_—Z-shaped assemblies were found. The energy decomposition analysis showed that the strongest interactions were found between blocks connected by N-H_par_⋯O=C_par_ and O-H_par_⋯O/N hydrogen bonds due to important coulombic factors. The dispersive energy becomes important for tail-to-tail (or head-to-tail) arranged paracetamol units and it prevails in case of stacking interactions between coformer molecules. The importance of dispersive forces increases with the size of the aromatic system of the coformer and the smallest is for the pyridine complex. However, for efficient interactions, the proper mutual orientation of both molecules must be assured. All these interactions affect paracetamol conformation and the position of its amide group described by the C2-N3-C3-C8 torsion angle changing in a broad range of angles. Therefore, two final remarks should be made: (i) if nitrogen is missing (e.g., nap), the hydrogen interactions are disabled for such coformer and (ii) if a nitrogen atom is present, the hydroxyl group is redirected and is involved in hydrogen bonding with the nitrogen atom of the coformer. The latter effect alters the hydrogen bond network and results in the formation of new supramolecular motifs in the paracetamol layers, namely $R_{4}^{4}$(22) in (**1**); $R_{6}^{4}$(20) and $R_{2}^{2}$(8) in (**2**); and $R_{2}^{2}$(8), $R_{4}^{2}$(12), and $R_{4}^{4}$(22) rings in (**3**) maintained by hydrogen bonds. Those data highlight the fact that further studies are needed to fully understand the driving forces governing the crystal network. CSP methods can possibly induce a breakthrough to achieve this goal.

## Data Availability

Crystallographic data have been deposited at The Cambridge Crystallographic Data Center via www.ccdc.cam.ac.uk/structures.

## Supplementary Materials

The following supporting information can be downloaded at: https://www.mdpi.com/article/10.3390/molecules29184437/s1, Figure S1. Thermal analysis for (**2**) (a) and (**3**) (b). Figure S2. IR spectra. Table S1. Intermolecular hydrogen bonds in (par)_2_∙(nap) (**1**). Table S2. C-H⋯π intermolecular interactions in (par)_2_∙(nap) (**1**). Table S3. Selected π-π intermolecular interactions in (par)_2_∙(nap) (**1**). Figure S3. (par)_2_∙(nap) (**1**)—the double paracetamol layer and nap layer. Figure S4. Hirshfeld surfaces and fingerprints in the crystal network of (par)_2_∙(nap) (**1**). Table S4. Intermolecular hydrogen bonds in (par)∙(quin) (**2**). Table S5. Selected π-π intermolecular interactions in (par)∙(quin) (**2**). Table S6. C-H⋯π and Y-X⋯π intermolecular interactions in (par)∙(quin) (**2**). Figure S5. Interaction in paracetamol and quinoline layers of (par)∙(quin) (**2**). Figure S6. Hirshfeld surfaces and fingerprints in the crystal network of (par)∙(quin) (**2**). Table S7. Intermolecular hydrogen bonds in (par)∙(acr) (**3**). Table S8. Selected π-π intermolecular interactions in (par)∙(acr) (**3**). Table S9. C-H⋯π intermolecular interactions in (par)∙(acr) (**3**). Figure S7. The acridine layer and chain of paracetamol in (par)∙(acr) (**3**). Figure S8. Hirshfeld surfaces and fingerprints in the crystal network of (par)∙(acr) (**3**). Figure S9. Energy of interactions in the crystal network of (par)∙(phe)_2_. Figure S10. Building block arrangement in (par)∙(phe)_2_. Figure S11. Building block arrangement in (par)_2_∙(pyr). Figure S12. Energy of interactions in the crystal network of (par)_2_∙(pyr). Table S10. The types of hydrogen bonds and energy of interactions. Table S11. The selected interactions between blocks. Figure S13. Histograms for C2-N3-C3-C8 torsion angle for par. Figure S14. Histograms for C2-N3-C3-C8 torsion angle for parH^+^. Table S12. XANES spectra for N K edge. Table S13. XANES spectra for O K edge. Figure S15. UV-Vis and fluorescence spectra for (**3**). Table S14. Crystal data and structure refinement for (**1**)–(**3**).
