# Supplementary material for: Driving Forces in the Formation of Paracetamol Cocrystals and Solvate with Naphthalene, Quinoline and Acridine
Source: Molecules. 2024 Sep 18;29(18):4437. doi: 10.3390/molecules29184437 (PMC11434482; doi:10.3390/molecules29184437)

checkCIF/PLATON report

Structure factors have been supplied for datablock(s) emiliab20Mo_auto

THIS REPORT IS FOR GUIDANCE ONLY. IF USED AS PART OF A REVIEW PROCEDURE FOR PUBLICATION, IT SHOULD NOT REPLACE THE EXPERTISE OF AN EXPERIENCED CRYSTALLOGRAPHIC REFEREE.

No syntax errors found. CIF dictionary Interpreting this report

Datablock: emiliab20Mo_auto

Bond precision:	C-C = 0.0021 A		Wavelength=0.71073
Cell:	a=12.4895(4)	b=15.7829(5)	c=8.8855(3)
	alpha=90	beta=103.290(3)	gamma=90
Temperature:	100 K		

	Calculated	Reported
Volume	1704.61(10)	1704.61(10)
Space group	P 21/c	P 21/c
Hall group	-P 2ybc	-P 2ybc
Moiety formula	C13 H9 N, C8 H9 N O2	(C13 H9 N), (C8 H9 N O2)
Sum formula	C21 H18 N2 O2	C21 H18 N2 O2
Mr	330.37	330.37
Dx,g cm-3	1.287	1.287
Z	4	4
Mu (mm-1)	0.084	0.084
F000	696.0	696.0
F000'	696.29	
h,k,lmax	17,22,12	17,22,12
Nref	5201	5188
Tmin,Tmax	0.991,0.994	0.677,1.000
Tmin'	0.980	

Correction method= # Reported T Limits: Tmin=0.677 Tmax=1.000
AbsCorr = MULTI-SCAN

Data completeness= 0.998 Theta(max)= 30.507

R(reflections)= 0.0525(3553) wR2(reflections)= 0.1301(5188)

S = 1.035 Npar= 234

The following ALERTS were generated. Each ALERT has the format
test-name_ALERT_alert-type_alert-level.
Click on the hyperlinks for more details of the test.

Alert level C

```
PLAT042_ALERT_1_C Calc. and Reported MoietyFormula Strings  Differ     Please Check
              Calc: C13 H9 N, C8 H9 N O2
              Rep.: (C13 H9 N), (C8 H9 N O2)
PLAT905_ALERT_3_C Negative K value in the Analysis of Variance ...     -0.373 Report
PLAT911_ALERT_3_C Missing FCF Refl Between Thmin & STh/L=    0.600          2 Report
                2  3  0,  -5  8  7,
```

Alert level G

```
PLAT883_ALERT_1_G No Info/Value for _atom_sites_solution_primary .     Please Do !
PLAT899_ALERT_4_G SHELXL2018 is Deprecated and Succeeded by SHELXL     2019/3 Note
PLAT910_ALERT_3_G Missing # of FCF Reflection(s) Below Theta(Min).          2 Note
                1  0  0,   1  1  0,
PLAT912_ALERT_4_G Missing # of FCF Reflections Above STh/L=  0.600          8 Note
PLAT941_ALERT_3_G Average HKL Measurement Multiplicity ..........         4.7 Low
PLAT969_ALERT_5_G The 'Henn et al.' R-Factor-gap value ..........       1.615 Note
              Predicted wR2: Based on SigI**2  8.06 or SHELX Weight 12.57
PLAT978_ALERT_2_G Number C-C Bonds with Positive Residual Density.         21 Info
```

0 **ALERT level A** = Most likely a serious problem - resolve or explain
0 **ALERT level B** = A potentially serious problem, consider carefully
3 **ALERT level C** = Check. Ensure it is not caused by an omission or oversight
7 **ALERT level G** = General information/check it is not something unexpected

2 ALERT type 1 CIF construction/syntax error, inconsistent or missing data
1 ALERT type 2 Indicator that the structure model may be wrong or deficient
4 ALERT type 3 Indicator that the structure quality may be low
2 ALERT type 4 Improvement, methodology, query or suggestion
1 ALERT type 5 Informative message, check

It is advisable to attempt to resolve as many as possible of the alerts in all categories. Often the minor alerts point to easily fixed oversights, errors and omissions in your CIF or refinement strategy, so attention to these fine details can be worthwhile. In order to resolve some of the more serious problems it may be necessary to carry out additional measurements or structure refinements. However, the purpose of your study may justify the reported deviations and the more serious of these should normally be commented upon in the discussion or experimental section of a paper or in the "special_details" fields of the CIF. checkCIF was carefully designed to identify outliers and unusual parameters, but every test has its limitations and alerts that are not important in a particular case may appear. Conversely, the absence of alerts does not guarantee there are no aspects of the results needing attention. It is up to the individual to critically assess their own results and, if necessary, seek expert advice.

Publication of your CIF in IUCr journals

A basic structural check has been run on your CIF. These basic checks will be run on all CIFs submitted for publication in IUCr journals (*Acta Crystallographica*, *Journal of Applied Crystallography*, *Journal of Synchrotron Radiation*); however, if you intend to submit to *Acta Crystallographica Section C* or *E* or *IUCrData*, you should make sure that full publication checks are run on the final version of your CIF prior to submission.

Publication of your CIF in other journals

Please refer to the *Notes for Authors* of the relevant journal for any special instructions relating to CIF submission.

PLATON version of 15/07/2024; check.def file version of 15/07/2024

Datablock emiliab20Mo_auto - ellipsoid plot

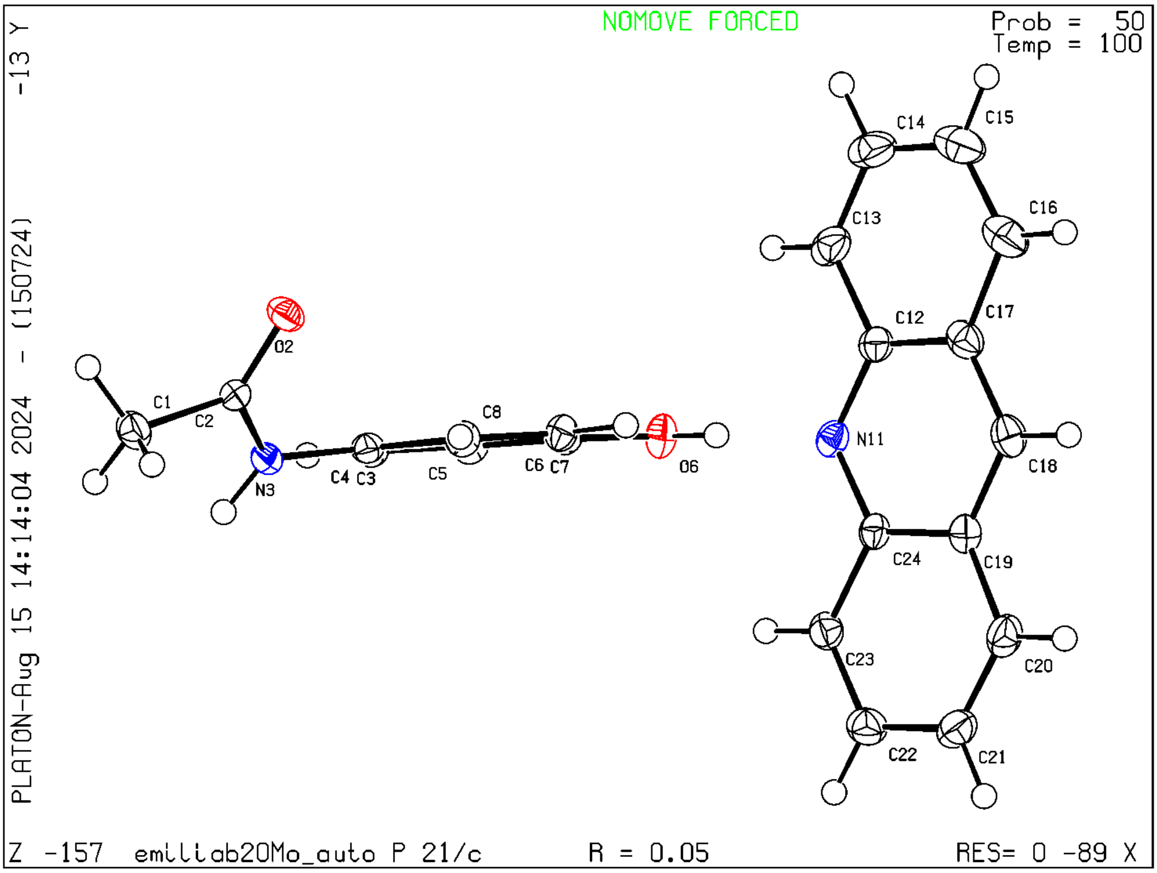

Supplement: Supplementary file 1 [file molecules-29-04437-s001.zip › molecules-3187401-SM/emiliab20Mo_auto-checkcif.pdf]
