# Supplementary material for: Driving Forces in the Formation of Paracetamol Cocrystals and Solvate with Naphthalene, Quinoline and Acridine
Source: Molecules. 2024 Sep 18;29(18):4437. doi: 10.3390/molecules29184437 (PMC11434482; doi:10.3390/molecules29184437)
Supplement: Supplementary file 1 [file molecules-29-04437-s001.zip › molecules-3187401-SM/muziol-molecules-revised4-Supplementary-Information.docx]

**Driving forces in the formation of paracetamol cocrystals and solvate with naphthalene, quinoline and acridine**

**Tadeusz M. Muzioł^1,^* and Emilia Bronikowska^1^**

^1^ Faculty of Chemistry, Nicolaus Copernicus University in Toruń, Gagarina 7, 87-100 Toruń, Poland; 307432@stud.umk.pl (E.B.)

***** Correspondence: tmuziol@umk.pl; Tel.: +48-56-6114976

**Supplementary Information**

**Contents**

Figure S1. Thermal analysis for (**2**) (a) and (**3**) (b). 3

Figure S2. IR spectra. 4

Table S1. Intermolecular hydrogen bonds in **(par)_2_∙(nap)** (**1**) found in PLATON. 6

Table S2. C-H∙∙∙π intermolecular interactions in **(par)_2_∙(nap)** (**1**) found in PLATON. 6

Table S3. Selected π-π intermolecular interactions in **(par)_2_∙(nap)** (**1**) found in PLATON. 6

Figure S3. **(par)_2_∙(nap)** (**1**) – the double paracetamol layer and nap layer. 7

Figure S4. Hirshfeld surfaces and fingerprints in the crystal network of **(par)_2_∙(nap)** (**1**) 7

Table S4. Intermolecular hydrogen bonds in **(par)∙(quin)** (**2**) found n PLATON. 10

Table S5. Selected π-π intermolecular interactions in **(par)∙(quin)** (**2**) found in PLATON. 10

Table S6. C-H∙∙∙π and Y-X∙∙∙π intermolecular interactions in **(par)∙(quin)** (**2**) found in PLATON. 10

Figure S5. Interaction in paracetamol and quinoline layers of **(par)∙(quin)** (**2**). 11

Figure S6. Hirshfeld surfaces and fingerprints in the crystal network of **(par)∙(quin)** (**2**). 12

Table S7. Intermolecular hydrogen bonds in **(par)∙(acr)** (**3**) found in PLATON. 15

Table S8. Selected π-π intermolecular interactions in **(par)∙(acr)** (**3**) found in PLATON. 15

Table S9. C-H∙∙∙π intermolecular interactions in **(par)∙(acr)** (**3**) found in PLATON. 15

Figure S7. The acridine layer and chain of paracetamol in **(par)∙(acr)** (**3**). 16

Figure S8. Hirshfeld surfaces and fingerprints in the crystal network of **(par)∙(acr)** (**3**) 17

Figure S9. Energy of interactions in the crystal network of **(par)∙(phe)_2_**. 20

Figure S10. Building block arrangement in **(par)∙(phe)_2_**. 20

Figure S11. Building block arrangement in **(par)_2_∙(pyr)**. 21

Figure S12. Energy of interactions in the crystal network of **(par)_2_∙(pyr)**. 21

Table S10. The types of hydrogen bonds and energy of interactions. 22

Table S11. The selected interactions between blocks. 22

Figure S13. Histograms for C2-N3-C3-C8 torsion angle for **par**. 23

Figure S14. Histograms for C2-N3-C3-C8 torsion angle for **parH^+^.**  23

Table S12. XANES spectra for N K edge. 24

Table S13. XANES spectra for O K edge. 24

Table S14. Crystal data and structure refinement for (**1**) – (**3**). 25


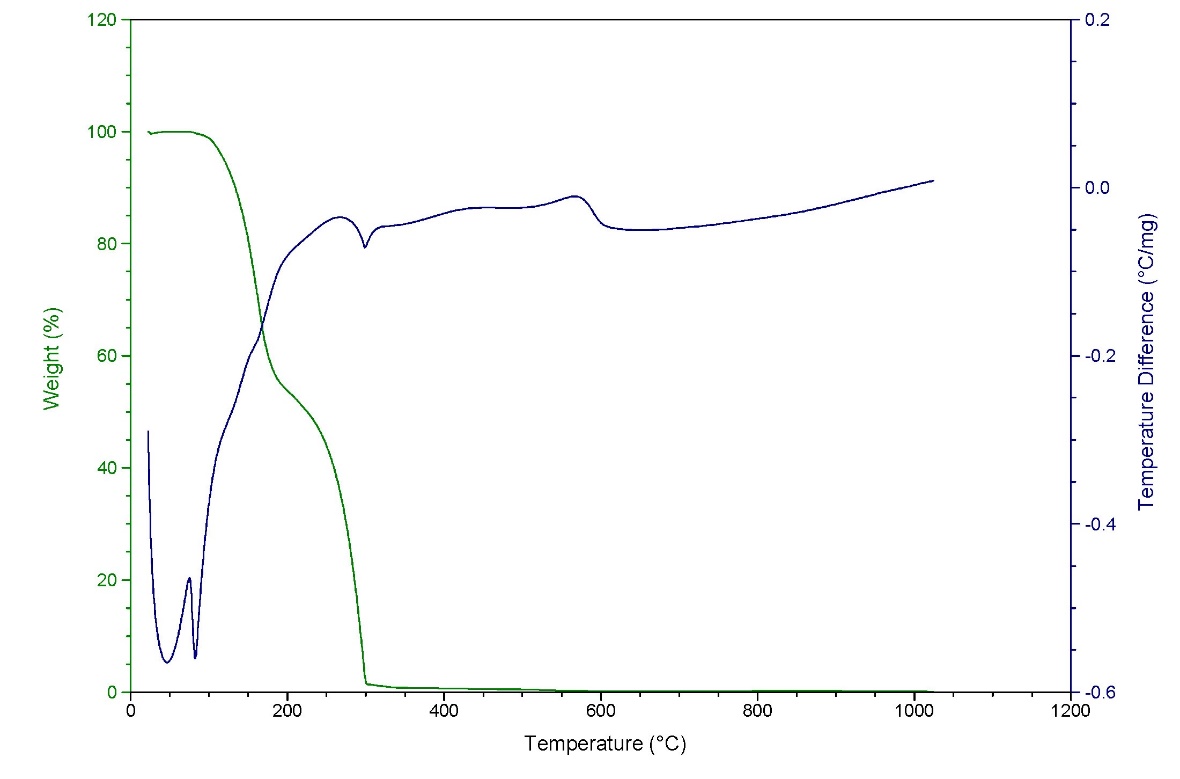


(a)


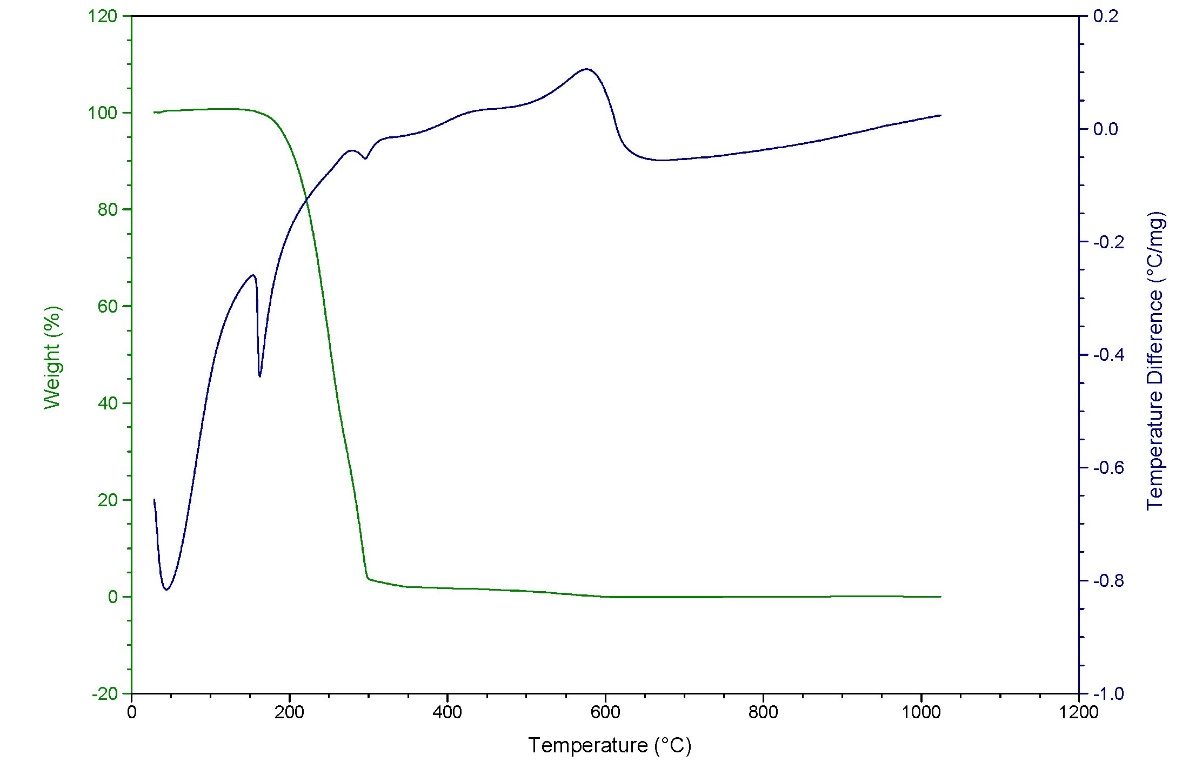


(b)

Figure S1. Thermal analysis for (**2**) (a) and (**3**) (b).

**IR spectra**


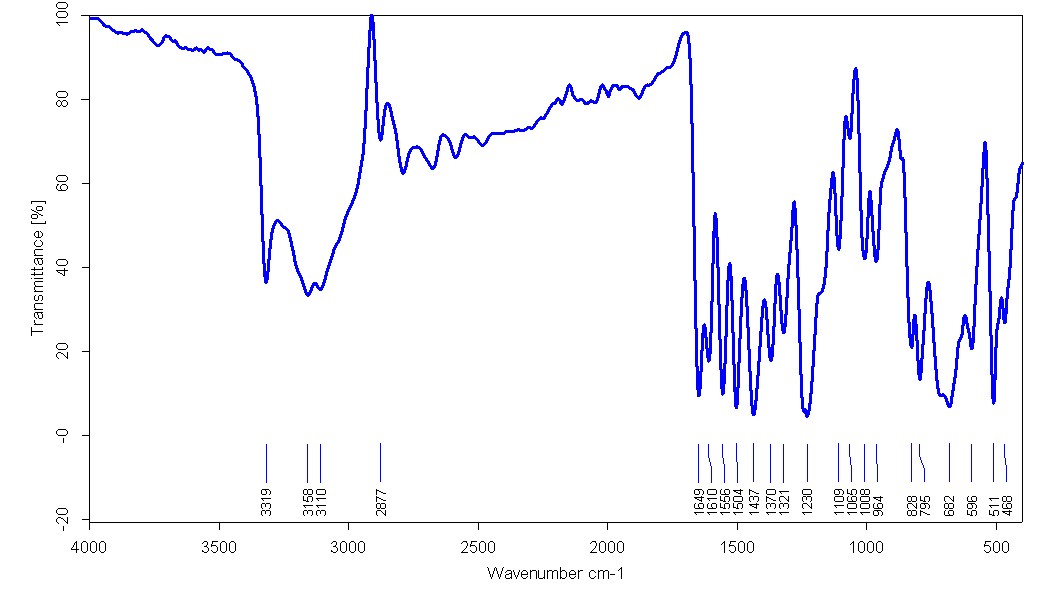


(**1**)


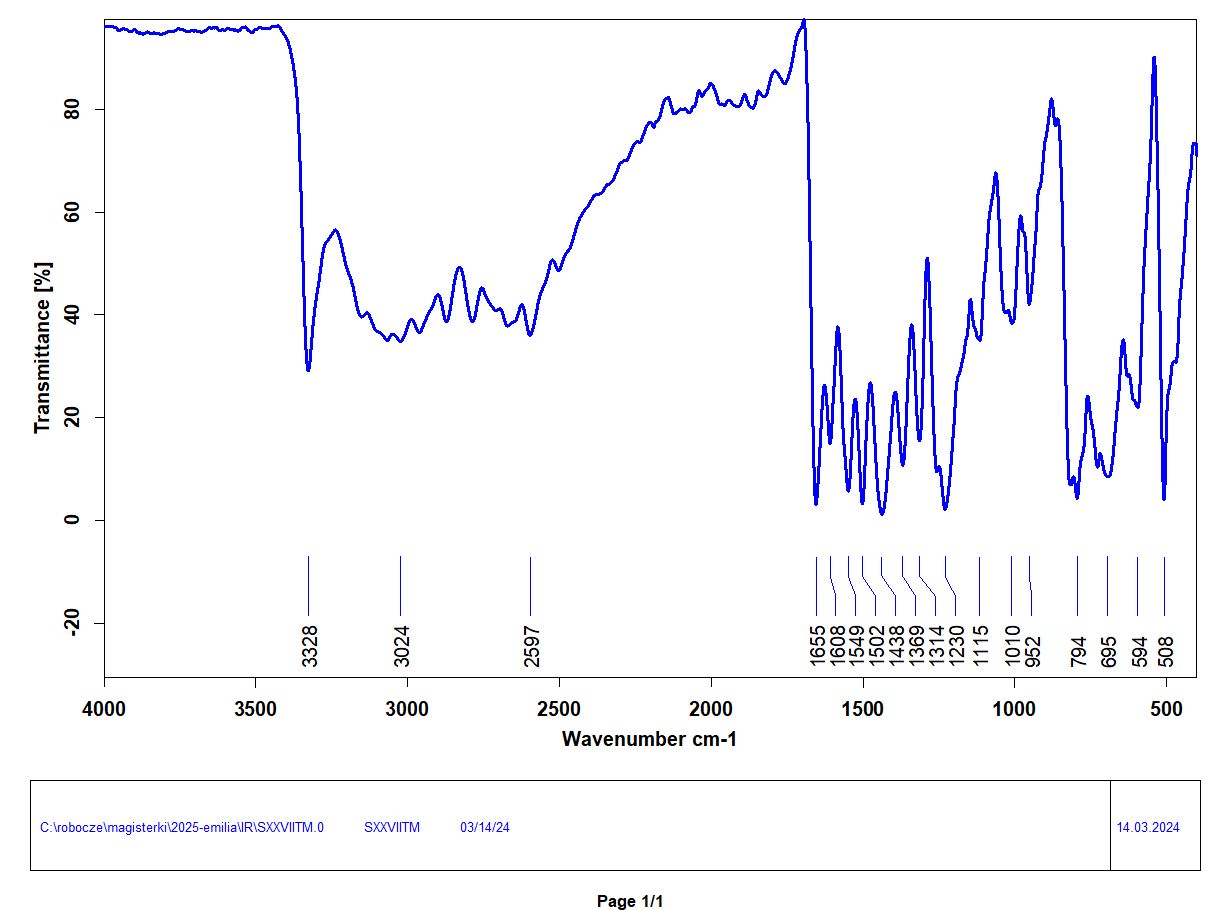


(**2**)


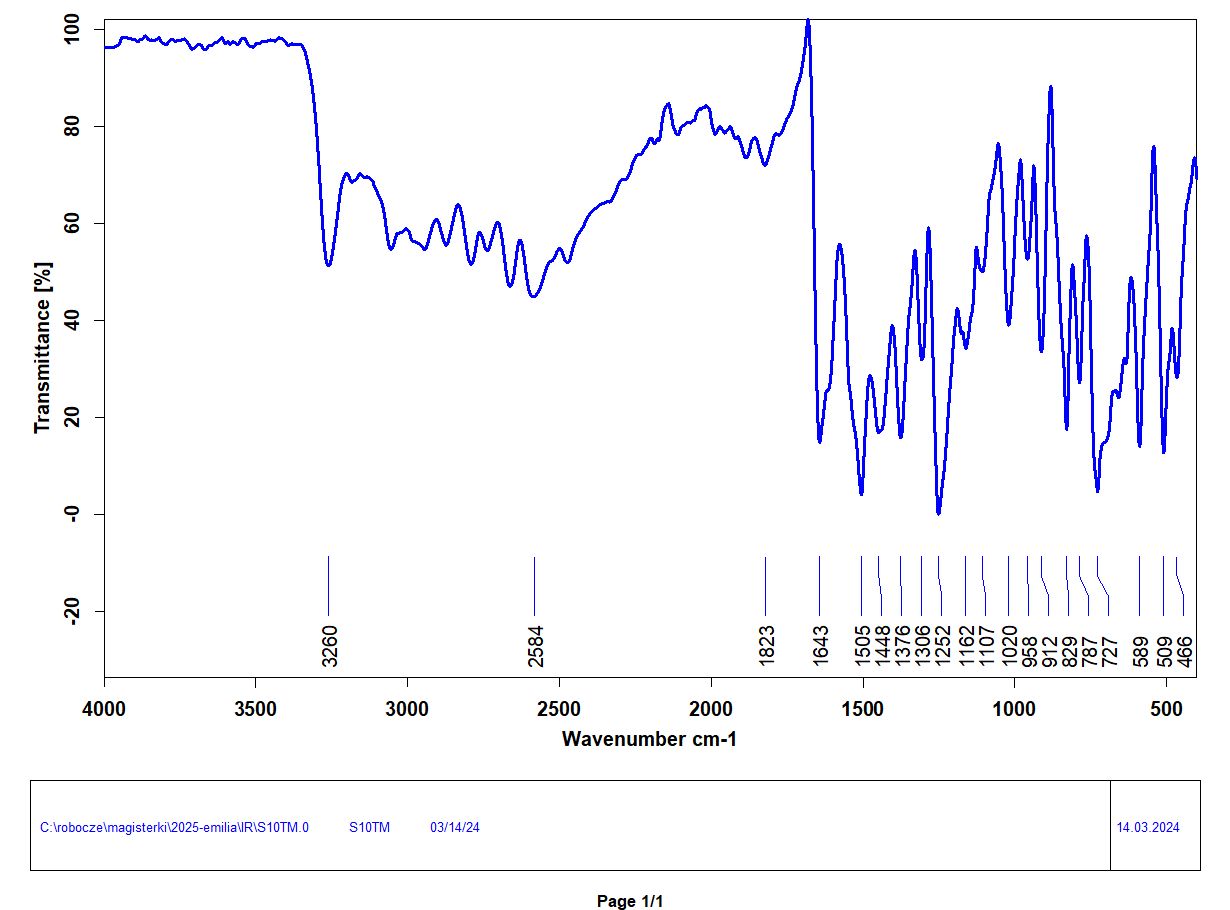


(**3**)

Figure S2. IR spectra recoded in ATR mode in the wavelength range of 4000 – 400 cm^-1^ for (**1**) – (**3**).

Intermolecular interactions in **(par)_2_∙(nap)** (**1**)

The selected interactions are given in the main text. Here, you can find more complete information about formed intermolecular interactions.

Table S1. Intermolecular hydrogen bonds in **(par)_2_∙(nap)** (**1**) found in PLATON.

| Donor atom [D-H] | Acceptor atom A [operator] | D-H [Å] | H∙∙∙A [Å] | D-H∙∙∙A angle [°] |
| --- | --- | --- | --- | --- |
| O3-H3 | O7 [x, -1+y, z] | 0.84 | 1.80 | 173 |
| N6-H6 | O3 [x,1/2-y,-1/2+z] | 0.92 | 2.02 | 168 |

Table S2. C-H∙∙∙π intermolecular interactions in **(par)_2_∙(nap)** (**1**) found in PLATON.

| Bond | ring [operator] | Distance of H to ring centroids [Å] | Angle between Cg∙∙∙H vector and ring normal [°] | X-H∙∙∙ring angle [°] |
| --- | --- | --- | --- | --- |
| C2-H2_par_ | C11_nap_[x, -1+y, z] | 2.95 | 12.50 | 52 |
| C2-H2_par_ | C11_nap_[1-x, 1-y, 1-z] | 2.95 | 12.50 | 52 |
| C8-H8_par_ | C3_par_[1-x, 1-y, 1-z] | 2.85 | 6.15 | 47 |
| C14-H14_nap_ | C3_par_[1-x, 1/2+y, 3/2-z] | 2.77 | 8.42 | 48 |

Table S3. Selected π-π intermolecular interactions in **(par)_2_∙(nap)** (**1**) found in PLATON.

| Interacting systems [operator] | Distance between ring centroids [Å] | Dihedral angle between planes of both rings [°] | Slippage [Å] |
| --- | --- | --- | --- |
| C3_par_∙∙∙C11_nap_[1-x, -1/2+y, 3/2-z] | 4.9849 | 52.44 | --- |
| C3_par_∙∙∙C11_nap_[x, 3/2-y, 1/2+z] | 4.9849 | 52.44 | --- |
| C11_nap_∙∙∙C3_par_[x, 1+y, z] | 4.9970 | 62.82 | --- |
| C11_nap_∙∙∙C3_par_[1-x, 1-y, 1-z] | 4.9970 | 62.82 | --- |


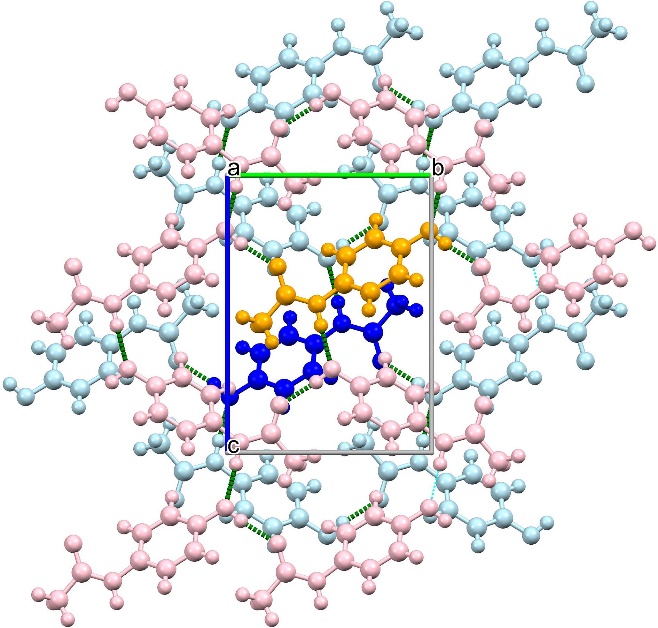

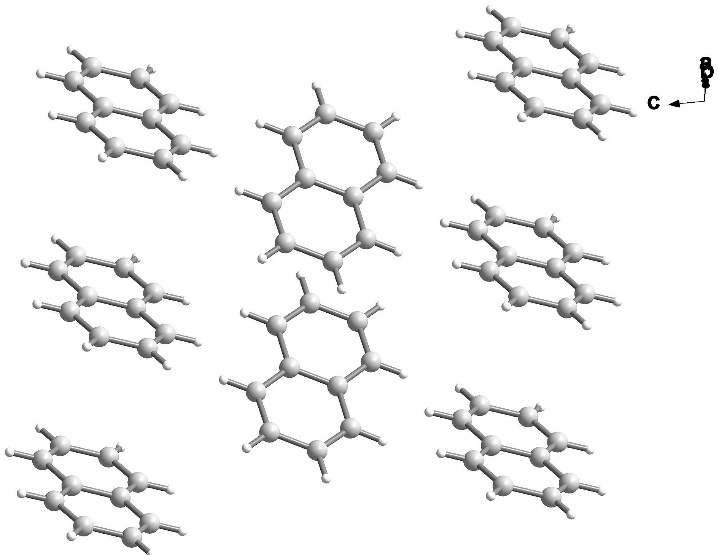


Figure S3. In **(par)_2_∙(nap)** (**1**), the superposed two paracetamol layers given in blue and pink showing head-to-tail oriented paracetamol molecules in both layers (left). In orange and blue are given two molecules from adjacent layers in a mode called head-to-tail in the manuscript. Naphthalene molecules are rather loosely arranged in the layer (right).


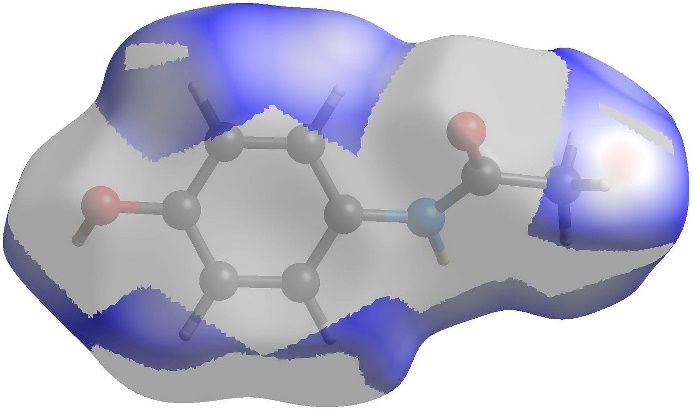

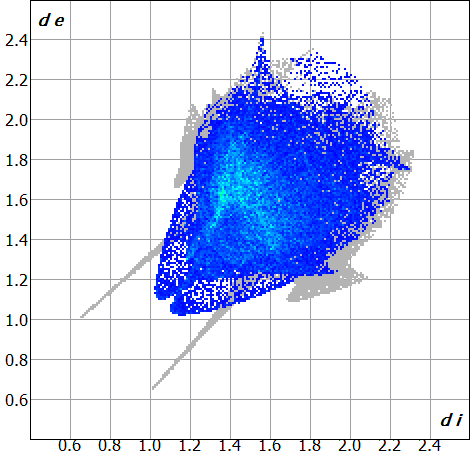


1. b.


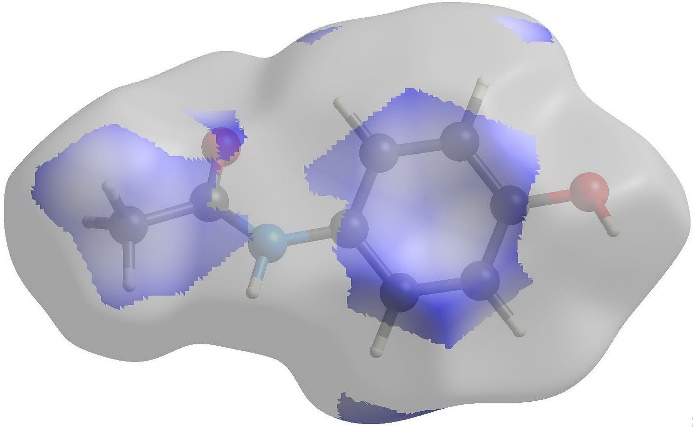

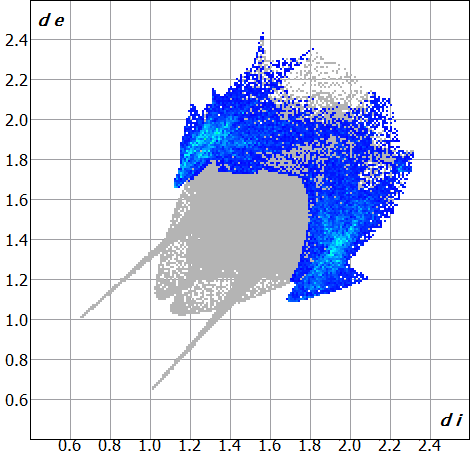


1. d.


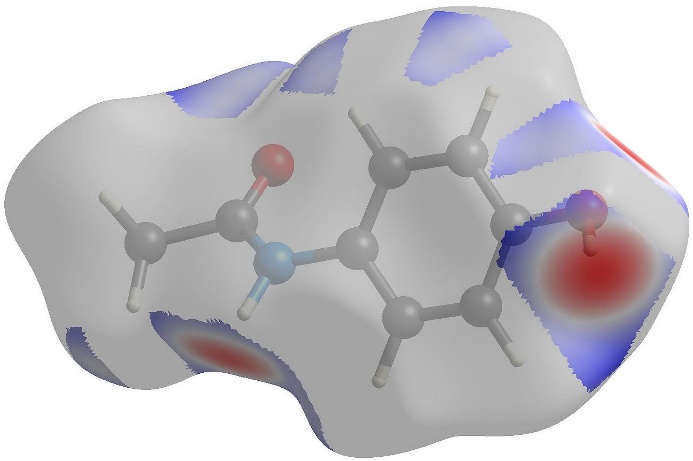

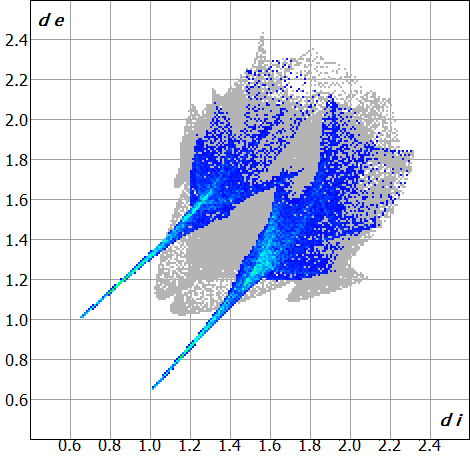


1. f.


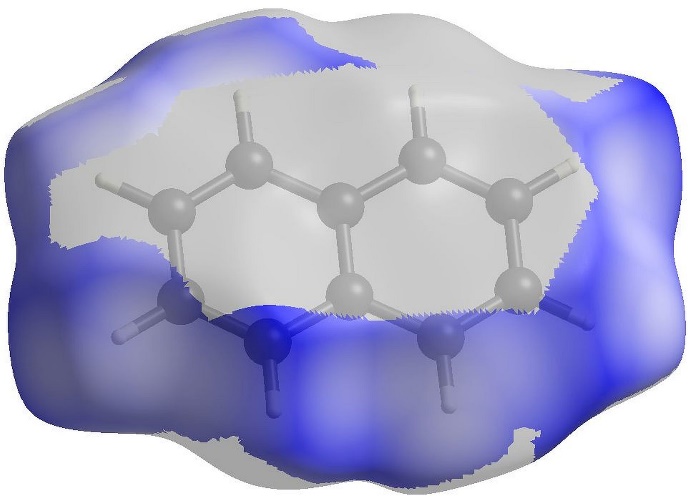

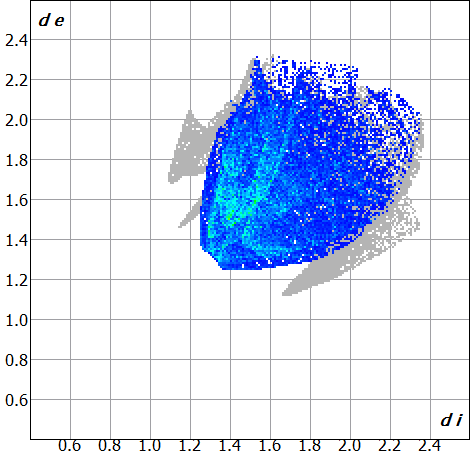


1. h.


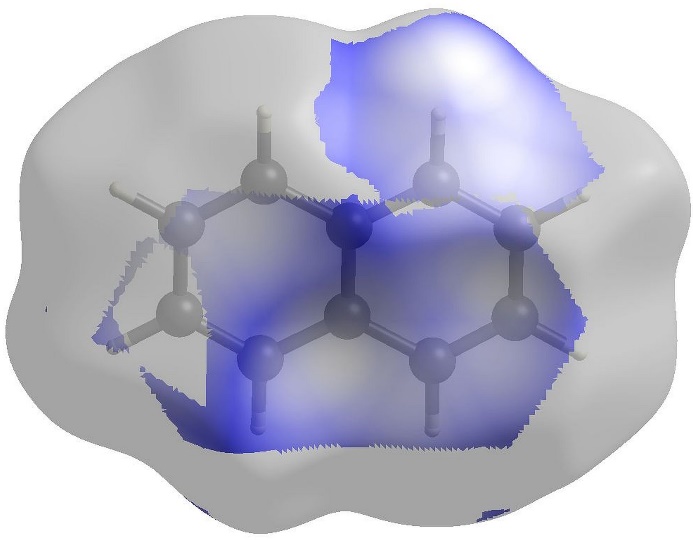

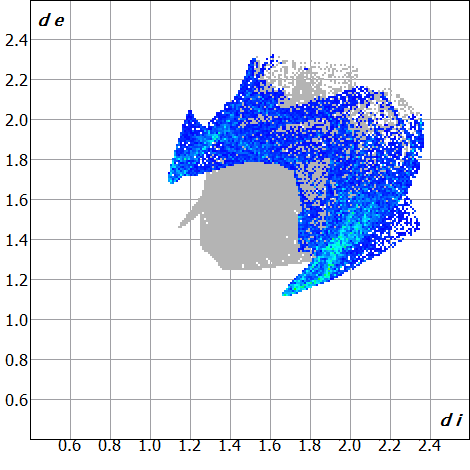


1. j.


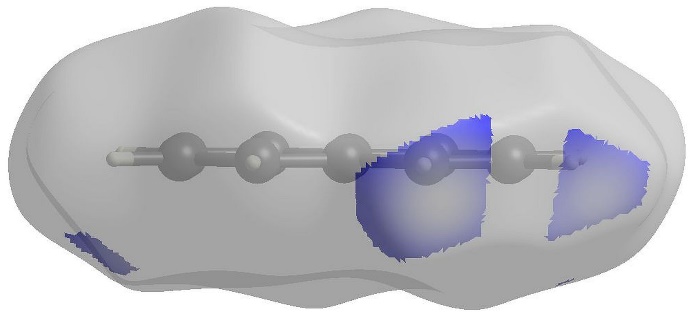

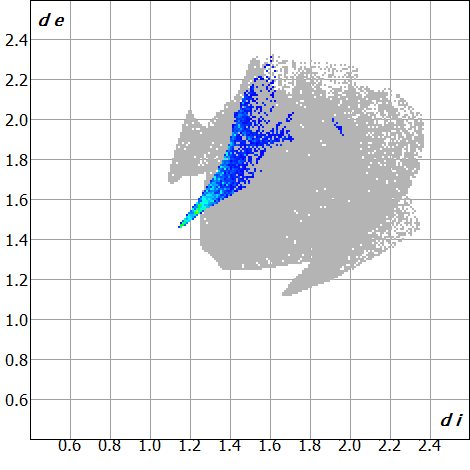


k. l

Figure S4. Hirshfeld surfaces (left) and fingerprints (right) of selected interactions created in the crystal network of **(par)_2_∙(nap)** (**1**): (a) and (b) for H⋯H (45.1%), (c) and (d) for H⋯C (27.6%), (e) and (f) for H∙∙∙O para (23.7%) in paracetamol molecules; (g) and (h) for H⋯H (52.5%), (i) and (j) for H∙∙∙C (36.4%) and (k) and (l) for H⋯O (6.7%) in naphthalene molecules. Colour coding for Hirshfeld surface and d_norm_ distance: red – for atoms with intermolecular distance shorter than the sum of van der Waals radii, white – for atoms with intermolecular distance being close to the sum of van der Waals radii and blue – for atoms with intermolecular distance longer than the sum of van der Waals radii. On the fingerprint, d_i_ and d_e_ are given in [Å]

Intermolecular interactions in **(par)∙(quin)** (**2**)

The selected interactions are given in the main text. Here, you can find more complete information about formed intermolecular interactions.

Table S4. Intermolecular hydrogen bonds in **(par)∙(quin)** (**2**) found n PLATON.

| Donor atom [D-H] | Acceptor atom A [operator] | D-H [Å] | H∙∙∙A [Å] | D-H∙∙∙A angle [°] |
| --- | --- | --- | --- | --- |
| N3-H3 | O2 [2-x, 1/2+y, -1/2-z] | 0.87 | 2.229 | 173.4 |
| O6-H6 | N11 [1-x, 1/2+y, 1/2-z] | 0.84 | 1.91 | 174 |
| C1-H1C | O2 [2-x, -y, -1-z] | 0.98 | 2.60 | 176 |

Table S5. Selected π-π intermolecular interactions in **(par)∙(quin)** (**2**) found in PLATON.

| Interacting systems [operator] | Distance between ring centroids [Å] | Dihedral angle between planes of both rings [°] | Slippage [Å] |
| --- | --- | --- | --- |
| C3_par_∙∙∙C3_par_[2-x, -y, -z] | 5.0789 | 0.00 | 4.120 |
| C3_par_∙∙∙C3_par_[x, 1/2-y, -1/2+z] | 5.6413 | 6.71 | 4.742 |
| C3_par_∙∙∙C3_par_[x, 1/2-y, 1/2+z] | 5.6413 | 6.71 | 4.541 |
| C3_par_∙∙∙N11_quin_[1-x, -y, -z] | 5.7225 | 67.60 | --- |
| C3_par_∙∙∙N11_quin_[x, y, -1+z] | 5.8553 | 68.56 | --- |
| N11_quin_∙∙∙C3_par_[x, -1/2-y, 1/2+z] | 5.5976 | 72.79 | --- |
| N11_quin_∙∙∙N11_quin_[x, -1/2-y, -1/2+z] | 5.5623 | 71.14 | --- |
| N11_quin_∙∙∙N11_quin_[1-x, -y, 1-z] | 3.5914 | 0.03 | 1.189 |
| N11_quin_∙∙∙N11_quin_[1-x, -1/2+y, 1/2-z] | 5.3511 | 71.53 | --- |

Table S6. C-H∙∙∙π and Y-X∙∙∙π intermolecular interactions in **(par)∙(quin)** (**2**) found in PLATON.

| Bond | ring [operator] | Distance of H/X to ring centroids [Å] | Angle between Cg∙∙∙H vector and ring normal [°] | X-H∙∙∙ring angle [°] |
| --- | --- | --- | --- | --- |
| C11-H11A_quin_ | C3_par_[1-x, -y, -z] | 2.47 | 1.63 | 62 |
| C2-O2_par_ | C3_par_[2-x, -y, -z] | 3.7892 | 18.63 | 18.10 |


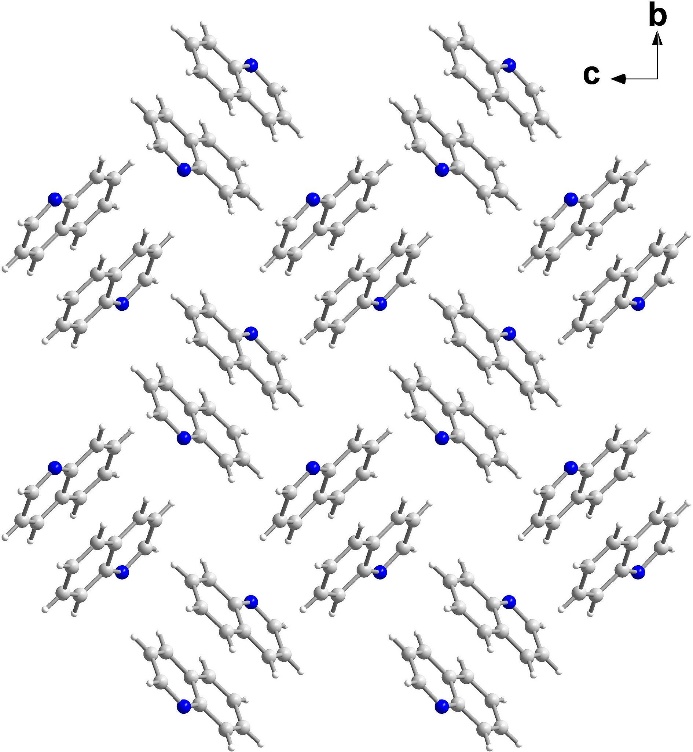

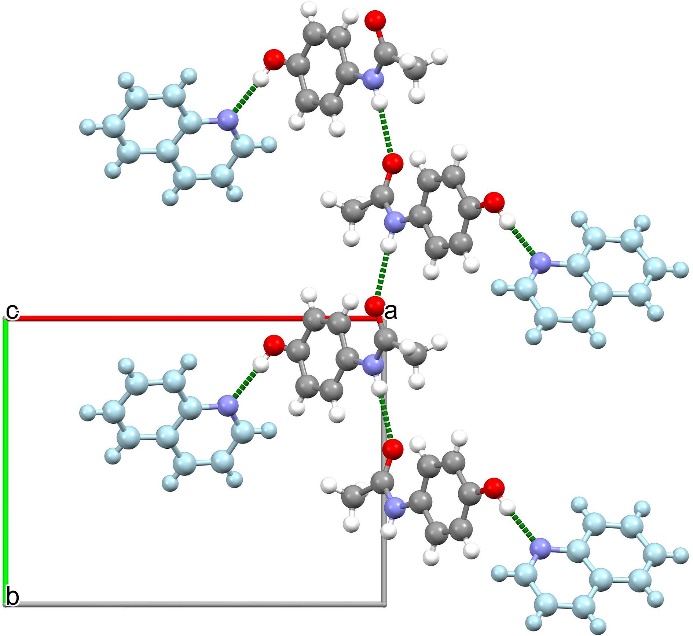


(a) (b)


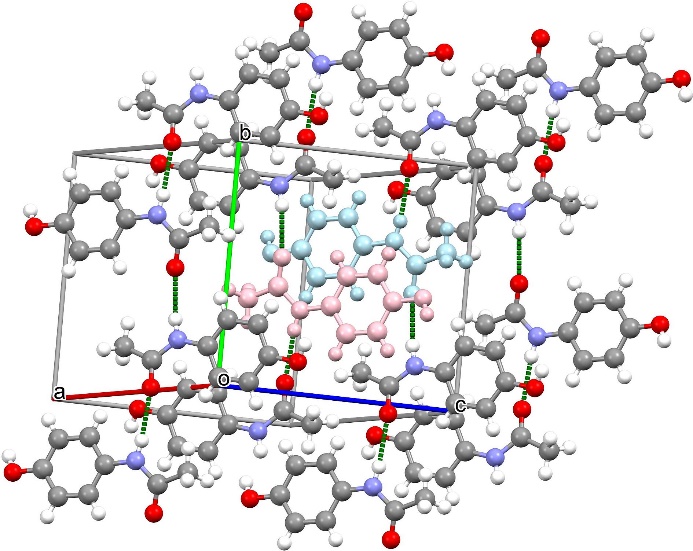


(c)

Figure S5. Interaction in paracetamol (a) and quinoline (b) layers of **(par)∙(quin)** (**2**). In the par layer the “peptide” hydrogen bonds are marked in green and C-H∙∙∙O in orange. The chain structure with marked quinoline molecules connected via O-H∙∙∙N hydrogen bonds is also presented. In the paracetamol layer only peptide hydrogen bonds are marked in green (c), In pink and cyan are given to molecules oriented in head-to-tail mode


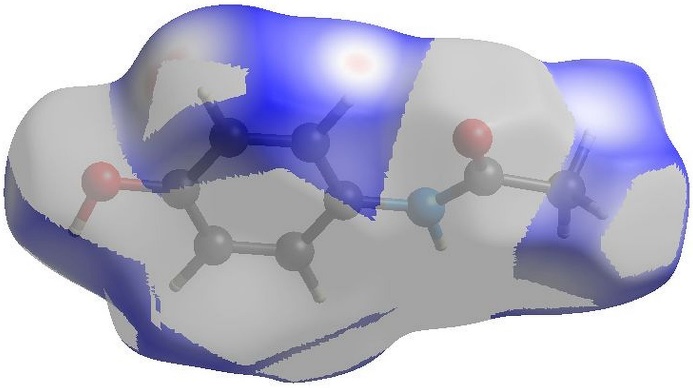

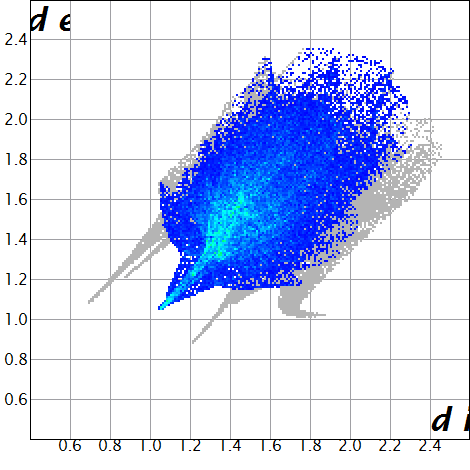


1. (b)


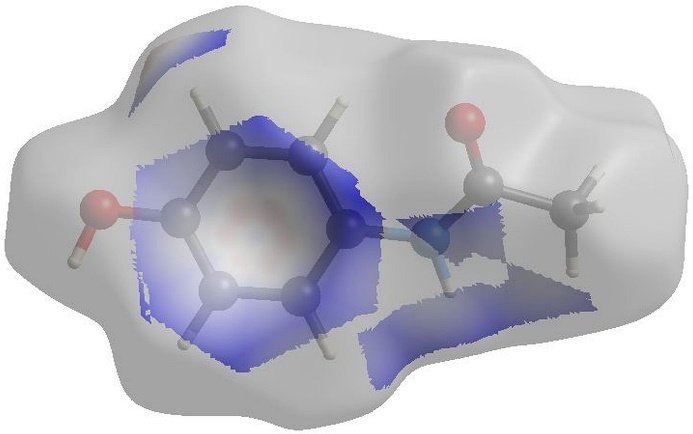

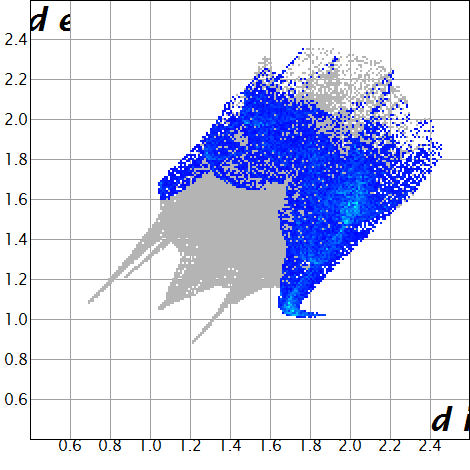


(c) (d)


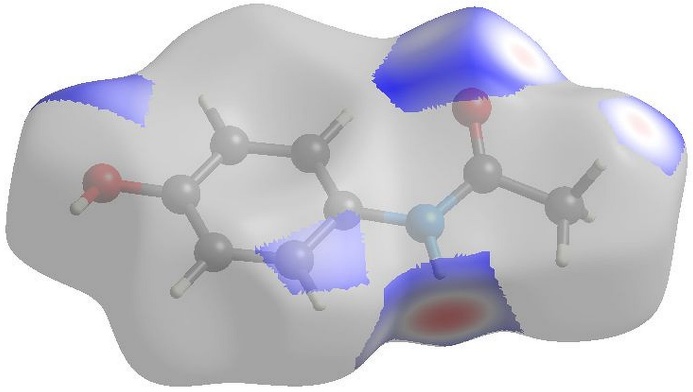

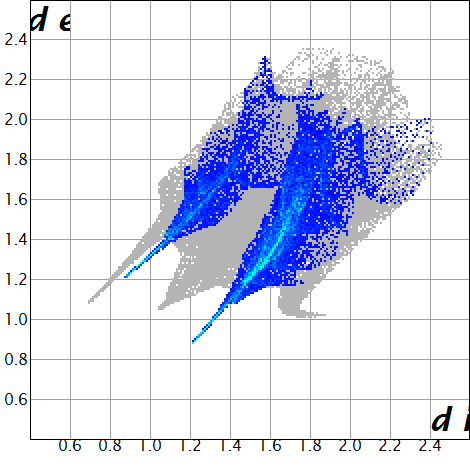


(e) (f)


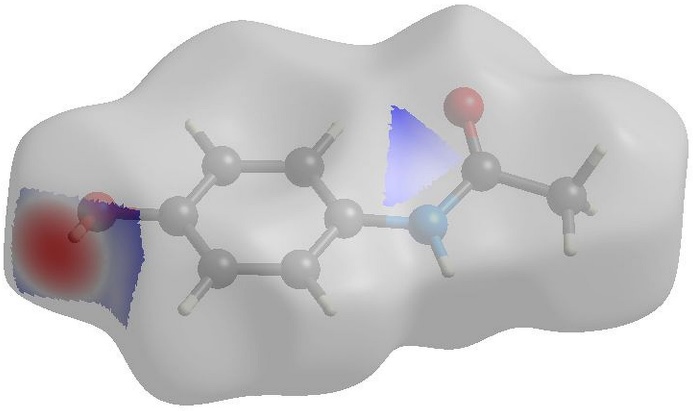

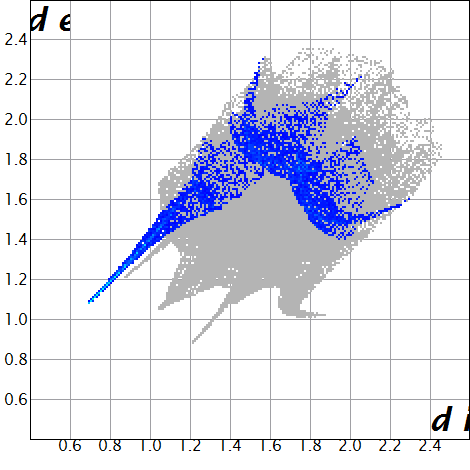


(g) (h)


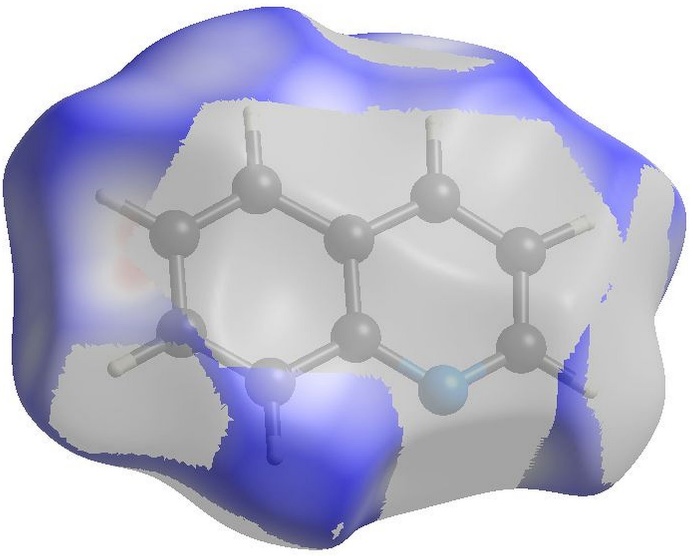

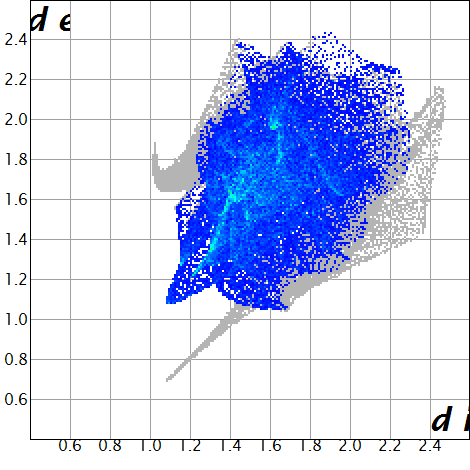


(i) (j)


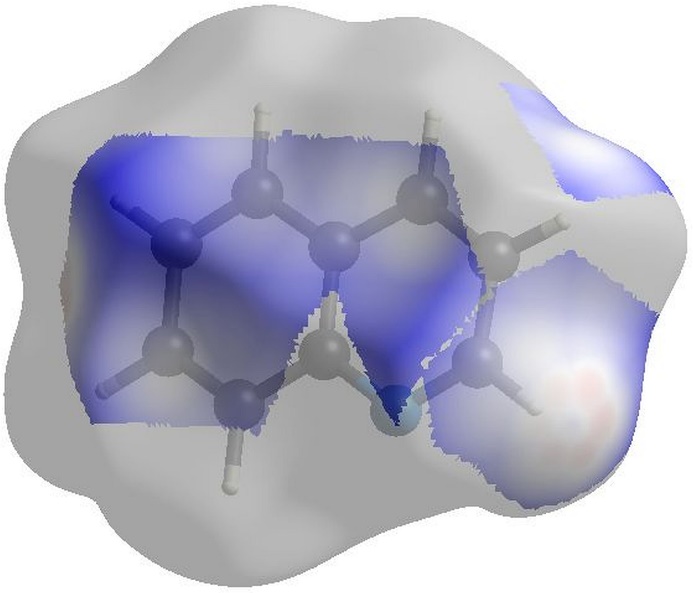

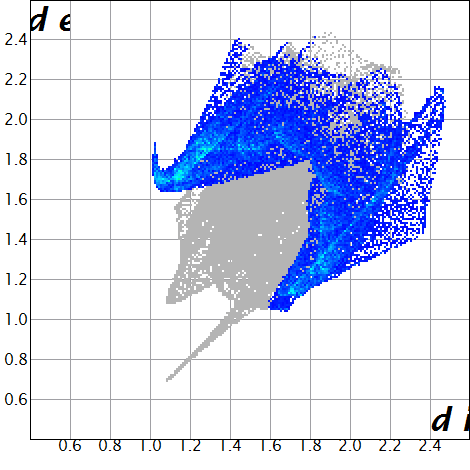


(k) (l)


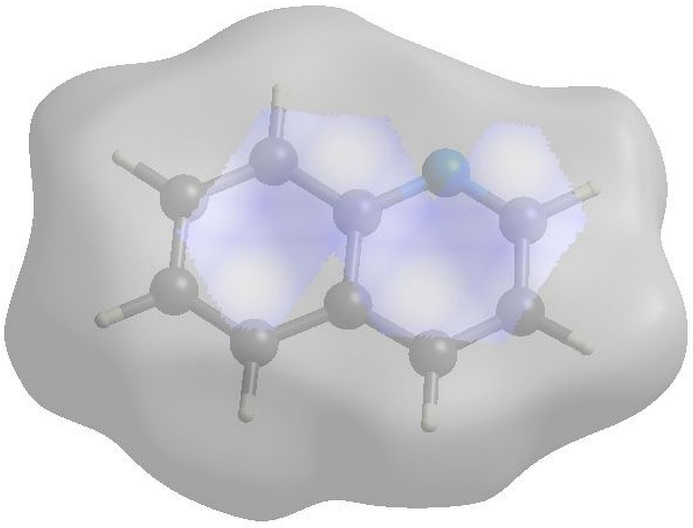

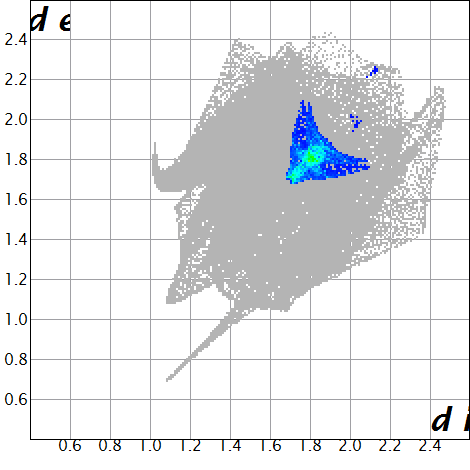


(m) (n)


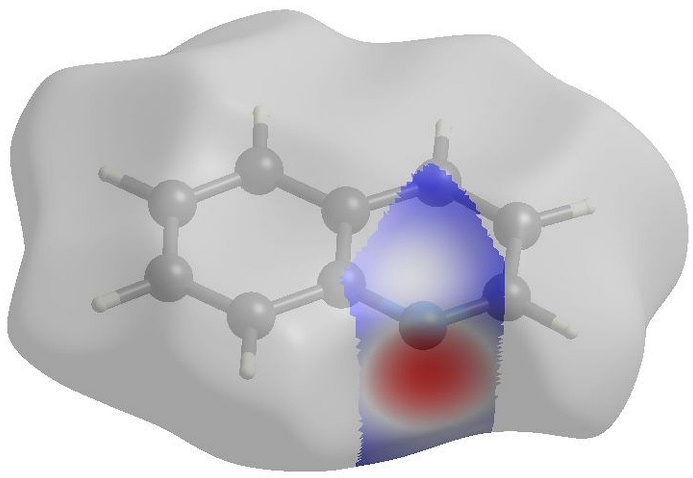

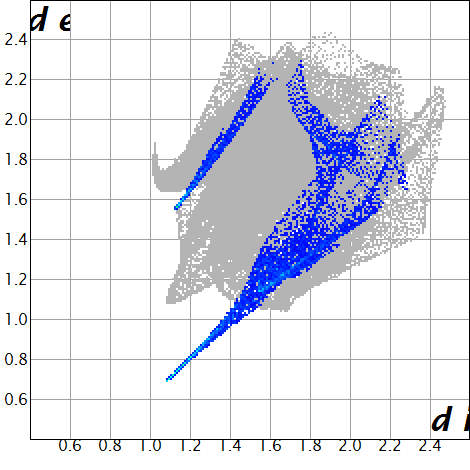


(o) (p)

Figure S6. Hirshfeld surfaces (left) and fingerprints (right) of selected interactions created in the crystal network of **(par)∙(quin)** (**2**): (a) and (b) for H⋯H (47.8%), (c) and (d) for H⋯C (20.6%), (e) and (f) for H∙∙∙O (21.6%), (g) and (h) for H∙∙∙N (6.7%) in paracetamol molecules; (i) and (j) for H⋯H (43.1%), (k) and (l) for H∙∙∙C (33.1%) and (m) and (n) for C⋯C (8.3%), (o) and (p) for H∙∙∙N (8.7%) in quinoline molecules.

Intermolecular interactions in **(par)∙(acr)** (**3**)

The selected interactions are given in the main text. Here, you can find more complete information about formed intermolecular interactions.

Table S7. Intermolecular hydrogen bonds in **(par)∙(acr)** (**3**) found in PLATON.

| Donor atom [D-H] | Acceptor atom A [operator] | D-H [Å] | H∙∙∙A [Å] | D-H∙∙∙A angle [°] |
| --- | --- | --- | --- | --- |
| N3-H3 | O2 [x, 3/2-y, -1/2+z] | 0.885 | 2.064 | 168.1 |
| O6-H6 | N11 [x, y, z] | 0.90 | 1.83 | 177 |
| C1-H1C | O6 [2-x,1/2+y,1/2-z] | 0.98 | 2.44 | 148 |
| C5-H5 | O2 [2-x, -1/2+y, 1/2-z] | 0.95 | 2.54 | 166 |
| C21-H21 | O2[1-x, 1-y, -z] | 0.95 | 2.65 | 170 |
| C22-H22 | O6 [1-x, 1-y, -z] | 0.95 | 2.48 | 154 |

Table S8. Selected π-π intermolecular interactions in **(par)∙(acr)** (**3**) found in PLATON.

| Interacting systems [operator] | Distance between ring centroids [Å] | Dihedral angle between planes of both rings [°] | Slippage [Å] |
| --- | --- | --- | --- |
| C3_par_∙∙∙N11_acr_[x, y, -1+z] | 4.8172 | 5.1 | --- |
| C3_par_∙∙∙ N11_acr_[1-x, 1-y, -z] | 5.3688 | 41.3 | --- |
| N11_acr_∙∙∙N11_acr_[1-x, 1-y, 1-z] | 3.9643 | 0.02 | 1.913 |
| N11_acr_∙∙∙N11_acr_[1-x, -y, -z] | 5.9924 | 53.85 | --- |

Table S9. C-H∙∙∙π intermolecular interactions in **(par)∙(acr)** (**3**) found in PLATON.

| Bond | ring [operator] | Distance of H/X to ring centroids [Å] | Angle between Cg∙∙∙H vector and ring normal [°] | X-H∙∙∙ring angle [°] |
| --- | --- | --- | --- | --- |
| C14-H14_quin_ | C3_par_[x, y, 1+z] | 2.84 | 15.33 | 63 |
| C16-H16_quin_ | N11_acr_[x, 3/2-y, 1/2+z] | 2.95 | 16.87 | 54 |


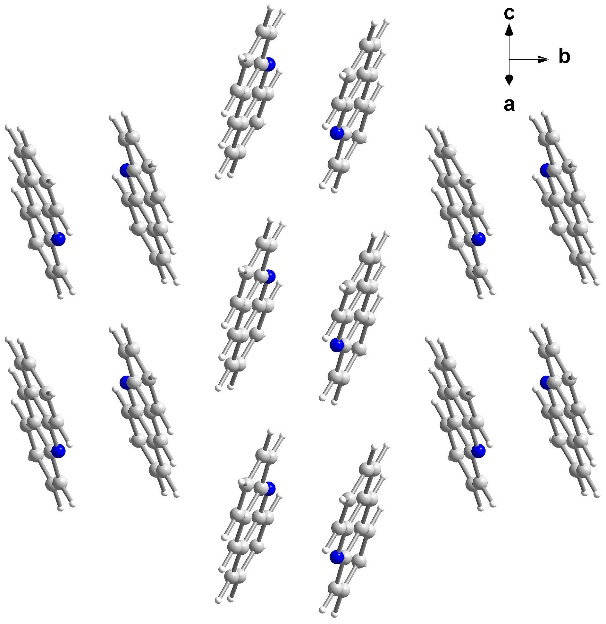

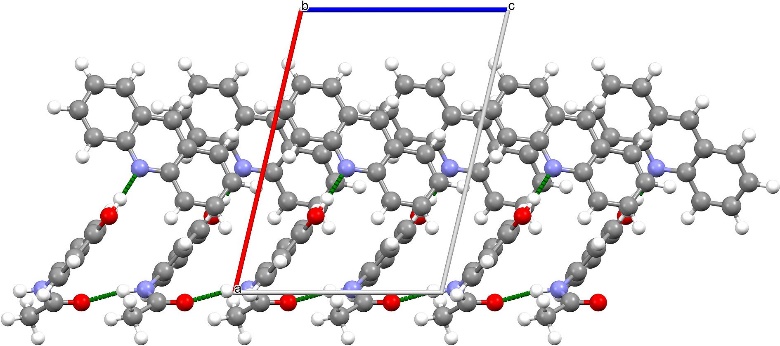


Figure S7. The acridine layer with dimers formed by stacking interactions capped by perpendicularly oriented adjacent acr molecules in **(par)∙(acr)** (**3**) (left) and chain of paracetamol connected by peptide N-H∙∙∙O bonds (right).


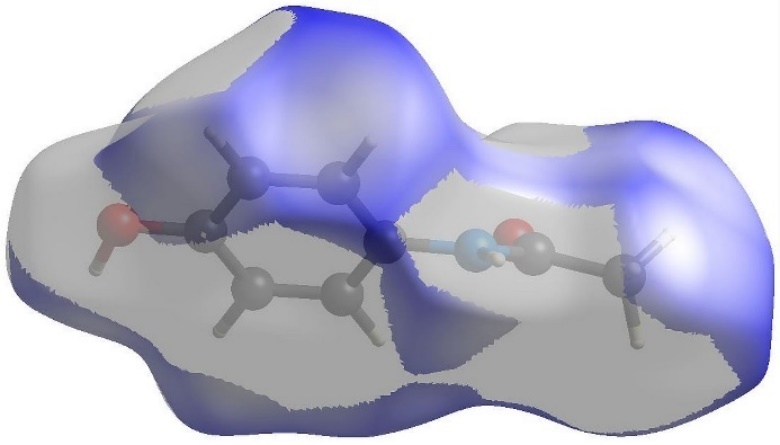

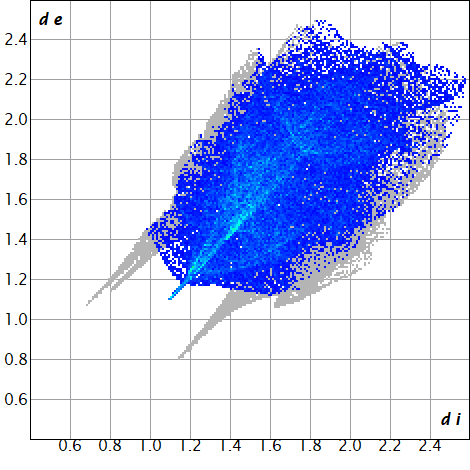


1. b.


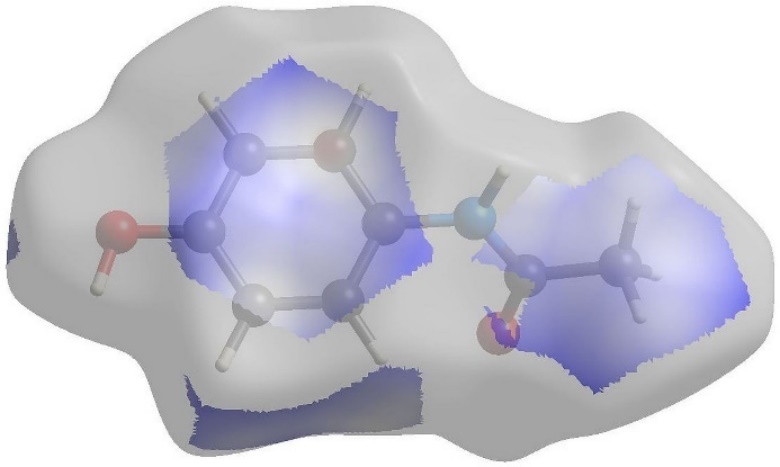

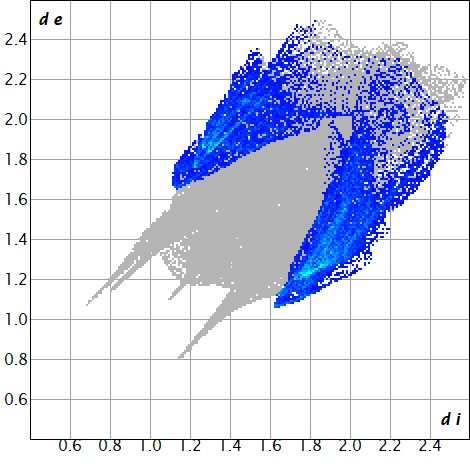


(c) (d)


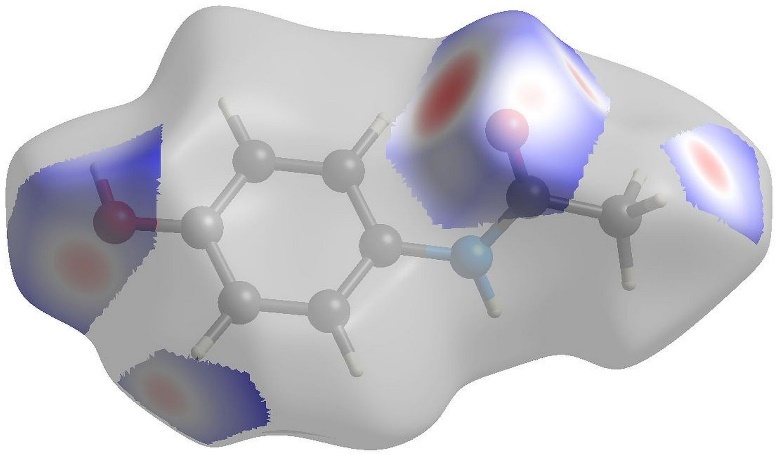

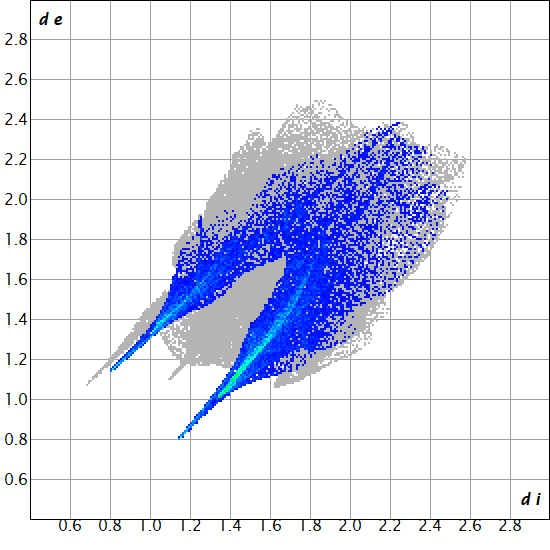


(e) (f)


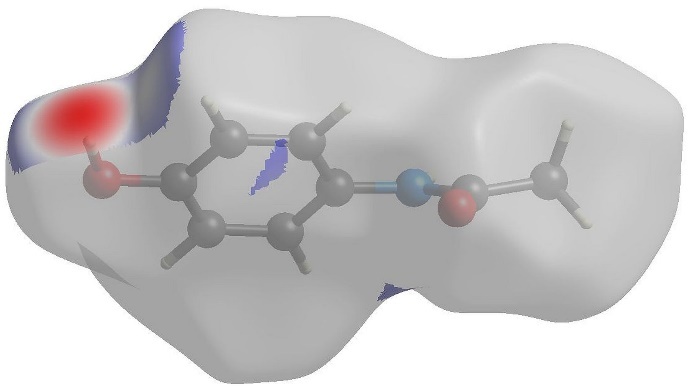

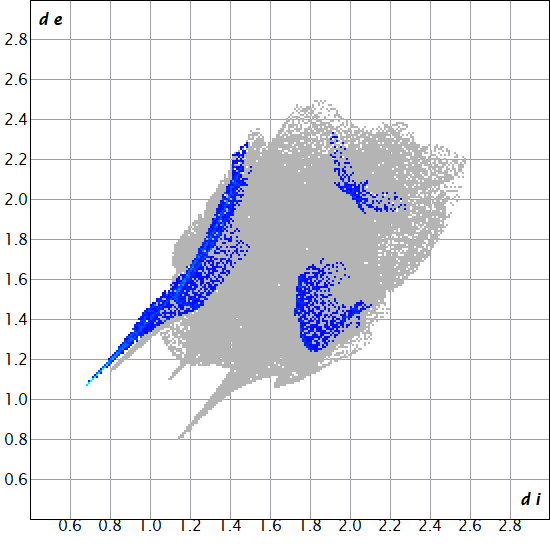


(g) (h)


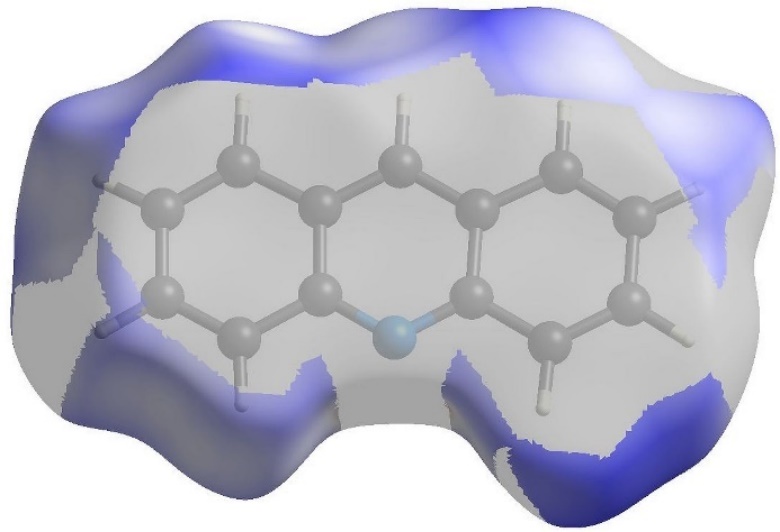

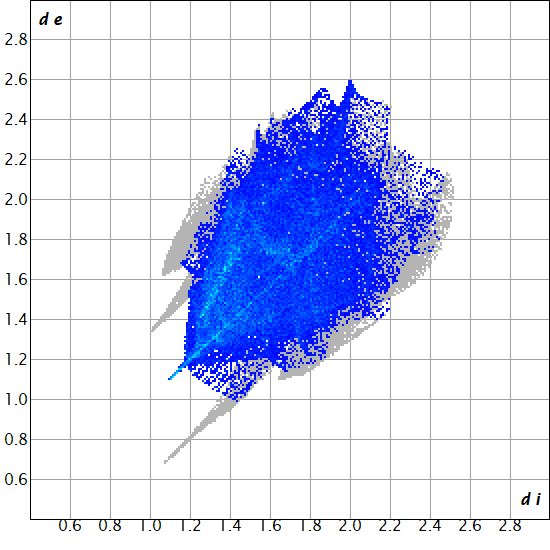


1. (j)


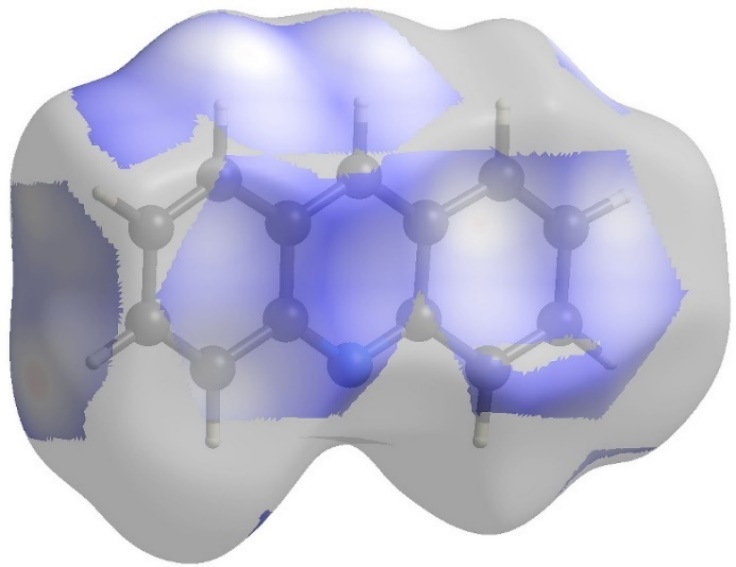

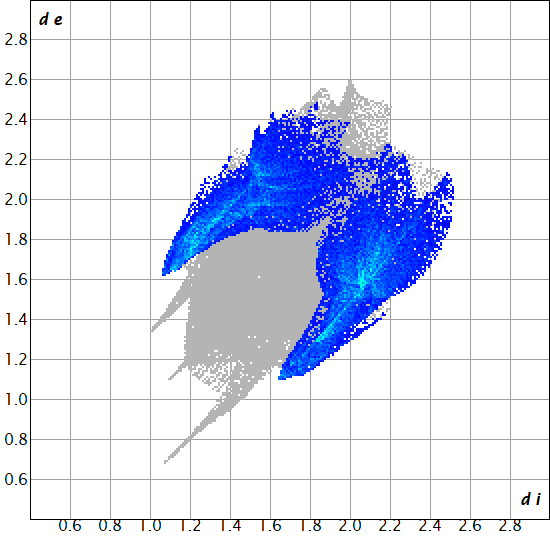


(k) (l)


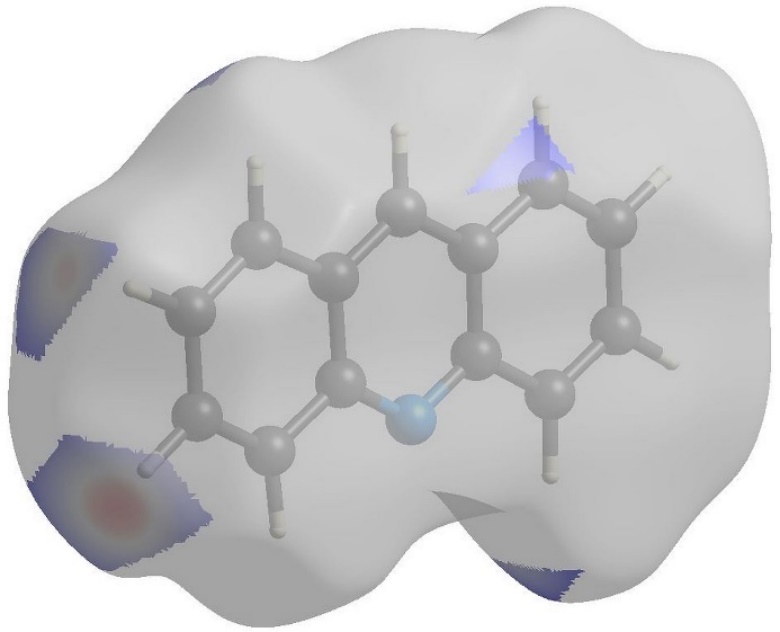

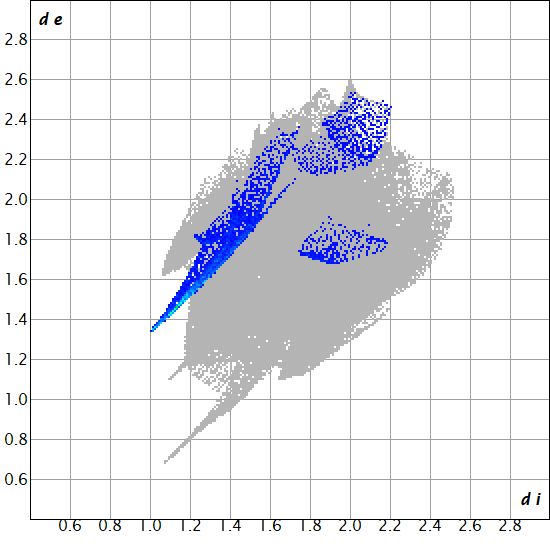


(m) (n)


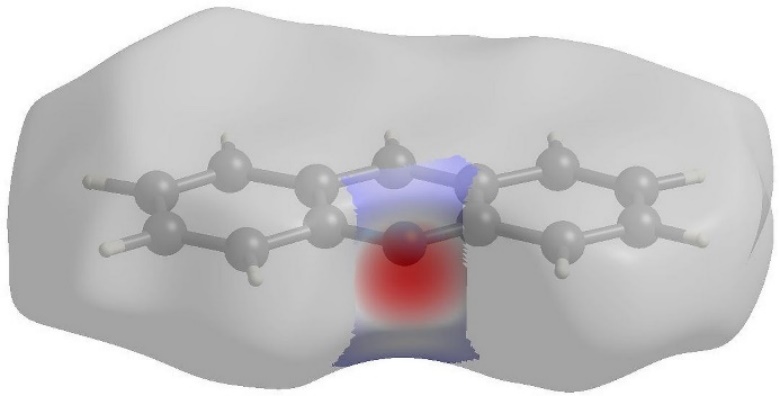

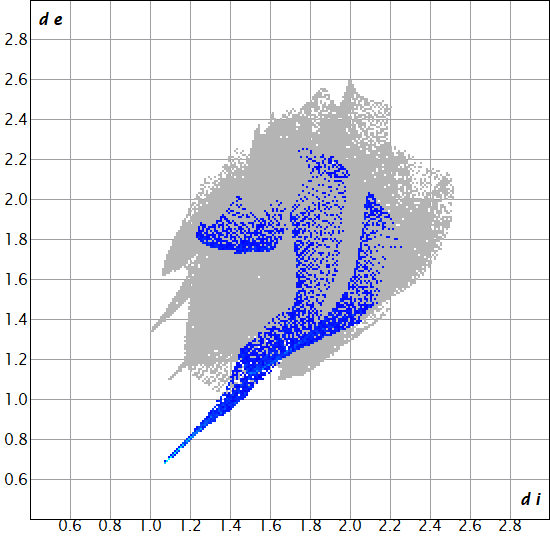


(o) (p)

Figure S8. Hirshfeld surfaces (left) and fingerprints (right) of selected interactions created in the crystal network of **(par)∙(acr)** (**3**): (a) and (b) for H⋯H (48.0%), (c) and (d) for H⋯C (23.4%), (e) and (f) for H∙∙∙O (22.8%), (g) and (h) for H∙∙∙N (5.0%) in paracetamol molecules; (i) and (j) for H⋯H (44.6%), (k) and (l) for H∙∙∙C (37.8%) and (m) and (n) for H⋯O (4.6%), (o) and (p) for H∙∙∙N (4.8%) in acridine molecules.


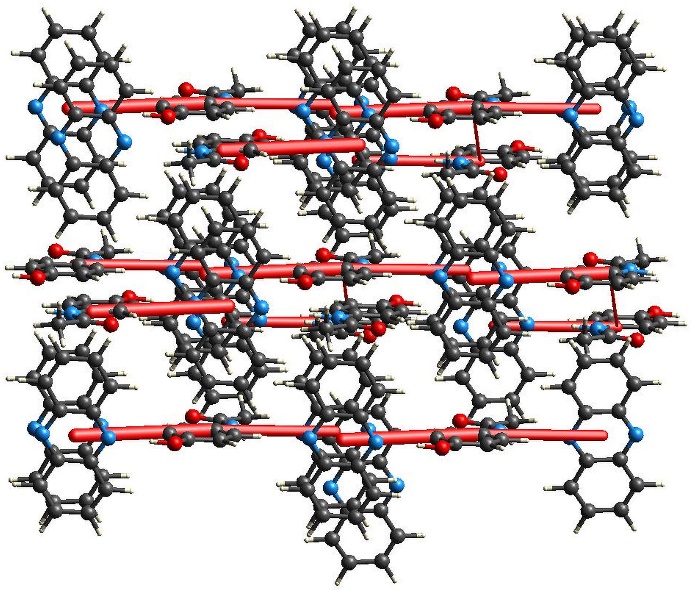

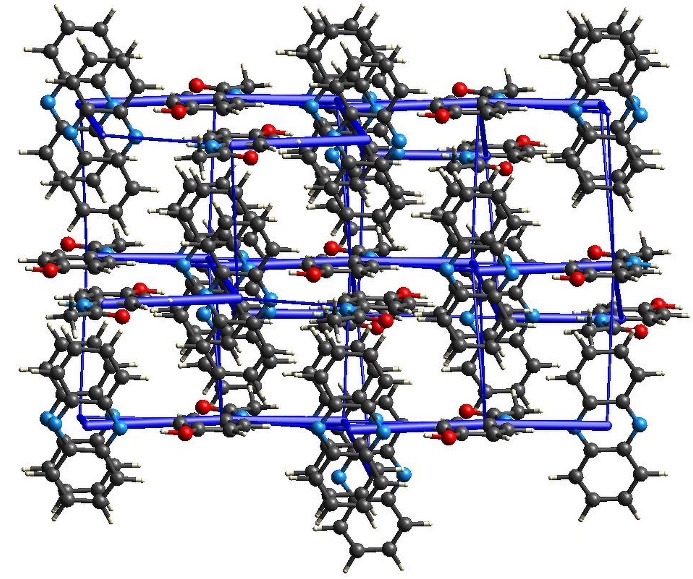

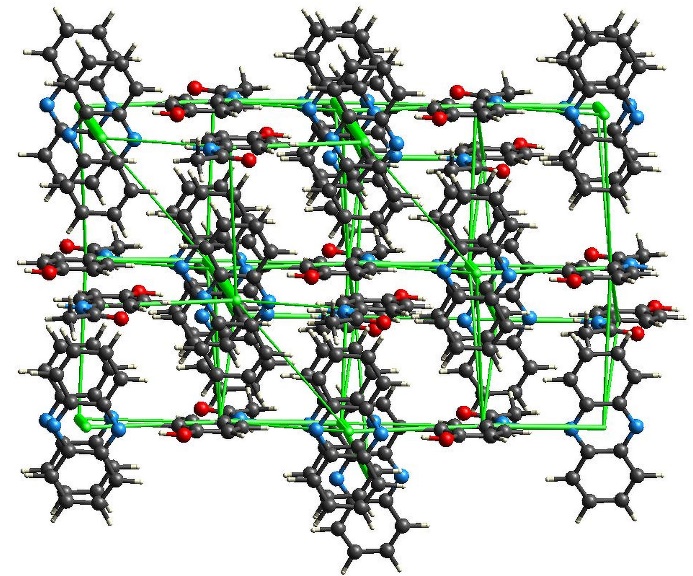


Figure S9. Energy of interactions between molecules in the crystal network of **(par)∙(phe)_2_** calculated in CrystalExplorer (red – electrostatic energy, green – dispersive energy and blue – total energy).


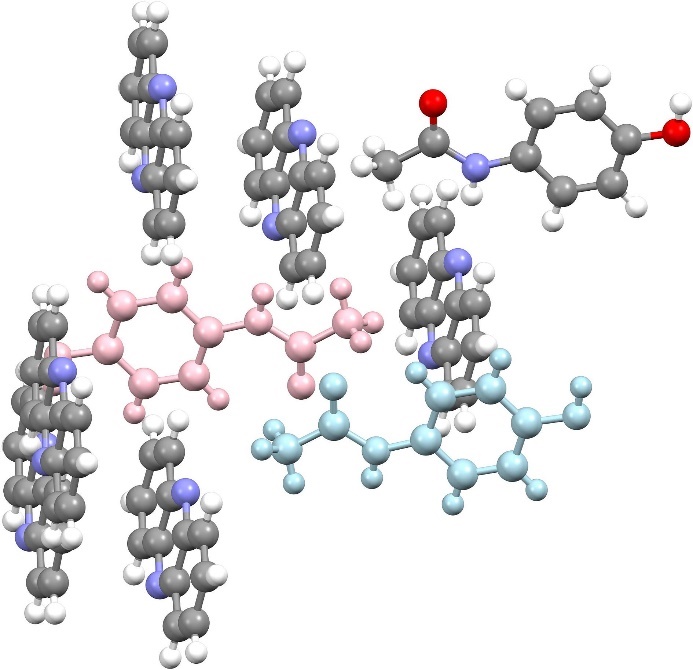


Figure S10. Building block arrangement in **(par)∙(phe)_2_**. Paracetamol molecules marked in pink and light blue are found in an orientation called in the text tail-to-tail.


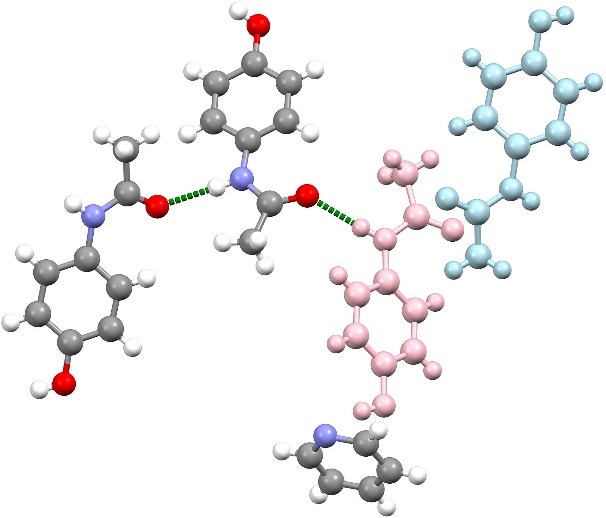


Figure S11. Building block arrangement in **(par)_2_∙(pyr)**. Paracetamol molecules marked in pink and light blue are found in an orientation called in the text tail-to-tail.


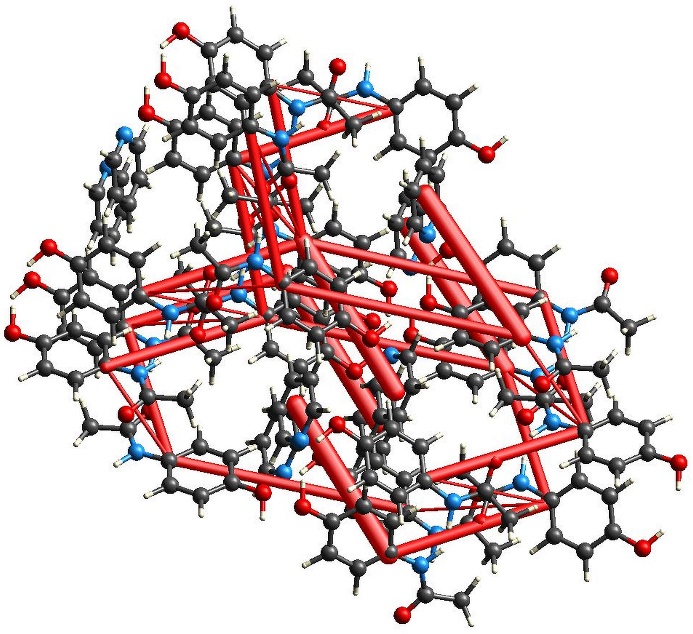

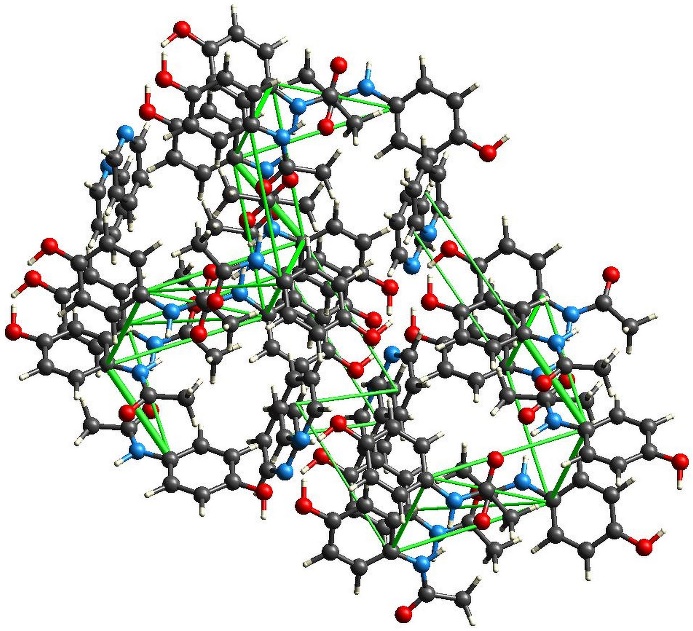


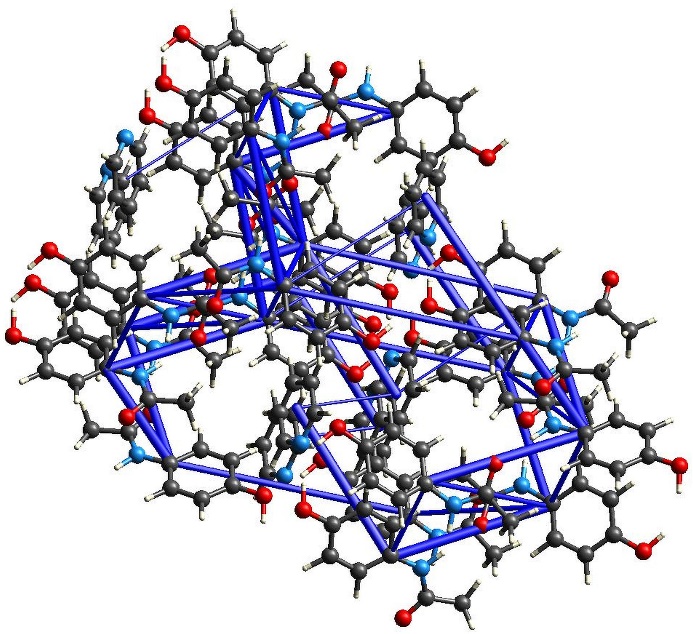


Figure S12. Energy of interactions between molecules in the crystal network of **(par)_2_∙(pyr)** calculated in CrystalExplorer (red – electrostatic energy, green – dispersive energy and blue – total energy).

Table S10. The types of hydrogen bonds and energy of interactions of blocks connected by those interactions are given. In the first column there is given a bond in paracetamol molecule with marked donor or acceptor in bold. In subsequent columns there are atoms (acceptor or donors of the hydrogen bonds) and in brackets energy of interactions between those moieties in kJ/mol. For (par)_2_∙(pyr) two values are given because there are two paracetamol molecules in the asymmetric unit (first line corresponds to A molecules and the second for B molecule).

| Atom in par | (**1**) | (**2**) | (**3**) | (par)_2_∙(pyr) | (par)∙(phe)_2_ |
| --- | --- | --- | --- | --- | --- |
| **O**-H (A) | N**H**_par_ (-27.2) | C-**H**_quin_ (-8.2) | C-**H**_3acr_ (-16.1) | O-**H**_par_ (-27.6)  C-**H**_pyr_ (-8.6) | C-**H**_phe_ (-8.7) |
| O-**H** (D) | C=**O**_par_ (-37.7) | **N**_quin_ (-45.0) | **N**_acr_ (-53.8) | **N**_pyr_ (-40.1)  **O**_par_ (-27.6) | **N**_phe_ (-45.8) |
| C=**O** (A) | O-**H**_par_ (-37.7) | N-**H**_par_ (-33.4) | N**H**_par_ (-46.8)  C-**H**_par_ (-16.1)  C-**H**_acr_ (-9.2) | N-**H**_par_ (-37.7)  N-**H**_par_ (-40.7) | C-H_phe_ (-8.9)  C-H_par_ (-23.2) |
| N-**H** (D) | **O**-H_par_ (-27.2) | C=**O**_par_ (-33.4) | C=**O**_par_ (-46.8) | C=**O**_par_ (-40.7)  C=**O**_par_ (-37.7) | **N**_phe_ (-43.8) |

A – acceptor, D – donor

Table S11. The selected interactions between blocks. In the first column is given a type of molecular arrangement which is usually mentioned in the text. In subsequent columns energy of interactions is given in kJ/mol.

| Molecule orientation | (**1**) | (**2**) | (**3**) | (par)_2_∙(pyr) | (par)∙(phe)_2_ |
| --- | --- | --- | --- | --- | --- |
| N-H∙∙∙O=C H-bond |  | -33.4 | -46.8 | -37.7  -40.7 |  |
| N-H∙∙∙N |  |  |  |  | -43.2 |
| O-H∙∙∙coformer |  | -45.0 | -53.8 | -40.1 | -45.8 |
| O-H∙∙∙par/H-O∙∙∙par | -37.7  -27.7 |  |  | -27.6 |  |
| π- π stacking | -5.8 | -27.0 | -30.4 | -12.8 | -30.9  -31.2 |
| π- π | -6.5 | -14.2 | -16.7 |  |  |
| par∙∙∙par (tail-to-tail or head-to-tail) | -30.2 | -26.5 |  | -40.1 | -23.2 |
| C-H_par_∙∙∙O-H_par_ | -10.1 | -19.2 | -16.1 |  |  |

**CSD structural analysis for structures with par and parH^+^ moieties.**

**Par**


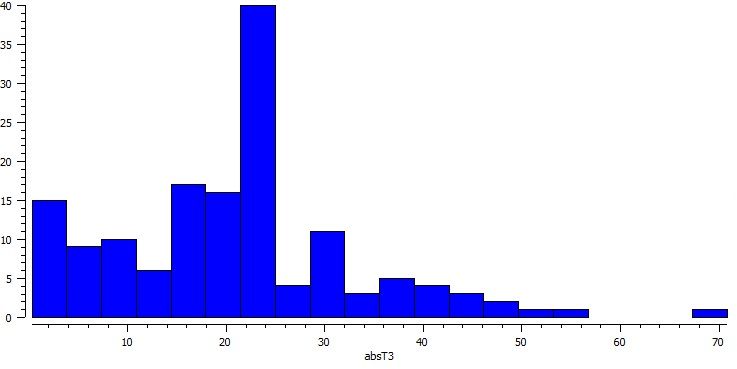


Figure S13. Histograms for C2-N3-C3-C8 torsion angle for **neutral paracetamol** structures (113 entries) (absT3 – absolute value of C2-N3-C3-C8).

**ParH^+^**


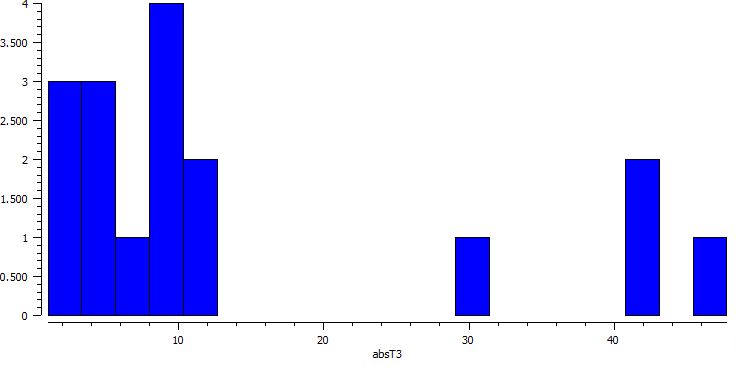


Figure S14. Histograms, scatterplot and heatmap corresponding to the scatterplot for structures with **paracetamol cation** (113 entries) (absT3 – absolute value of C2-N3-C3-C8, Dist1 – C-Nar, Dist2 – C-NH, Dist3 – C=O).

Table S12. XANES spectra for N K edge. Features in the range of 398 – 405 eV correspond to 1s🡪π* and features above 405 eV correspond to 1s🡪σ* transitions [6]. calculated (par)_2_∙(nap) (blue), calculated (par)∙(quin) (light brown), calculated

| Compound (TEY detector) | Energy peak for L_3_ and L_2_ [eV] | Intensity | Compound (TFY detector) | Energy peak for L_3_ and L_2_ [eV] | Intensity |
| --- | --- | --- | --- | --- | --- |
| (par)_2_∙(nap) | 398.6 401.5 403.1 406.6 409.2 | 0.25 0.87 0.78 1.15 1.22 | (par)_2_∙(nap) | 398.7 401.5 403.1 406.5 409.3 | 0.11 0.73 0.80 1.09/ 1.19/ |
| (par)∙(quin) | 399.0 401.5 403.1 406.6 409.4 | 0.28 0.78 0.84 1.15/ 1.25 | (par)∙(quin) | 398.8 401.5 403.1 406.4 409.4 | 0.09 0.73 0.80 1.07 1.19/ |
| (par)∙(acr) | 398.2 401.3 403.0 407.0 409.4 | 0.70 0.88 0.80 1.37 1.26 | (par)∙(acr) | 398.3 401.4 403.0 406.9 409.2 | 0.67 0.58 0.56 1.03 1.14/ |

Table S13. XANES spectra for O K edge. Low energy features are related to 1s🡪π* and high energy features correspond to 1s🡪σ* transitions.

| Compound (TEY detector) | Energy peak for L_3_ and L_2_ [eV] | Intensity | Compound (TFY detector) | Energy peak for L_3_ and L_2_ [eV] | Intensity |
| --- | --- | --- | --- | --- | --- |
| (par)_2_∙(nap) | 532.2 535.3 539.9 | 1.77 0.82 2.21 | (par)_2_∙(nap) | 532.3 535.3 540.0 | 1.07 0.73 1.45 |
| (par)∙(quin) | 532.3 535.5 539.8 | 1.12 0.71 1.69 | (par)∙(quin) | 532.3 535.5 540.1 | 1.27 0.73 1.46 |
| (par)∙(acr) | 532.1 535.2 539.9 | 1.96 0.86 1.99 | (par)∙(acr) | 532.3 535.3 540.0 | 1.15 0.63 1.57 |

Table S14. Crystal data and structure refinement for (par)_2_∙(nap) (**1**), (par)∙(quin) (**2**) and (par)∙(acr) (**3**).

| Identification code | (para)_2_∙naph (**1**) | (para)∙quin (**2**) | (para)∙acr (**3**) |
| --- | --- | --- | --- |
| Empirical formula | C_26_ H_26_ N_2_ O_4_ | C_17_ H_16_ N_2_ O_2_ | C21 H18 N2 O2 |
| Formula weight | 430.49 | 280.32 | 330.389 |
| Temperature | 100(2) K | 100(2) K | 100.00(10) K |
| Wavelength | 1.54184 Å | 1.54184 Å | 0.71073 Å |
| Crystal system | Monoclinic | Monoclinic | Monoclinic |
| Space group | P2**_1_**/c | P2**_1_**/c | P2**_1_**/c |
| Unit cell dimensions [Å] and [°] | a = 11.0038(5) α = 90 | a = 13.9317(2) α = 90 | a = 12.4895(4) α = 90° |
|  | b = 8.4799(3) β = 100.814(3) | b = 10.1886(3) β = 104.6086(16) | b = 15.7829(5) β = 103.290(3)°. |
|  | c = 11.7078(3) γ = 90 | c = 10.12290(16) γ = 90 | c = 8.8855(3) γ = 90°. |
| Volume [Å^3^] | 1073.07(7) Å3 | 1390.44(5) | 1704.61(10) |
| Z | 2 | 4 | 4 |
| Density (calculated) [Mg/m^3^] | 1.332 | 1.339 | 1.287 |
| Absorption coefficient [mm^-1^] | 0.730 mm-1 | 0.717 | 0.084 |
| F(000) | 456 | 592 | 696 |
| Crystal size [mm^3^] | 0.150 x 0.100 x 0.080 mm3 | 0.230 x 0.180 x 0.090 | 0.240 x 0.090 x 0.070 |
| Theta range for data collection [°] | 4.090 to 80.078°. | 3.278 to 74.489. | 2.581 to 30.507°. |
| Index ranges | -13<=h<=13 -10<=k<=10 -14<=l<=10 | -17<=h<=14 -12<=k<=12 -12<=l<=12 | -17<=h<=16 -22<=k<=22 -11<=l<=12 |
| Reflections collected | 11388 | 10814 | 24620 |
| Independent reflections | 2271 [R(int) = 0.0496] | 2848 [R(int) = 0.0126] | 5188 [R(int) = 0.0994] |
| Completeness to theta | 67.684° 99.9 % | 67.684° 100.0 % | 25.242° 99.9 % |
| Absorption correction | Semi-empirical from equivalents | Semi-empirical from equivalents | Semi-empirical from equivalents |
| Max. and min. transmission | 1.00000 and 0.75180 | 1.00000 and 0.93189 | 1.00000 and 0.67697 |
| Refinement method | Full-matrix least-squares on F2 | Full-matrix least-squares on F^2^ | Full-matrix least-squares on F2 |
| Data / restraints / parameters | 2271 / 0 / 151 | 2848 / 0 / 196 | 5188 / 0 / 234 |
| Goodness-of-fit on F2 | 1.095 | 1.039 | 1.035 |
| Final R indices [I>2sigma(I)] | R1 = 0.0499, wR2 = 0.1340 | R1^a^ = 0.0327, wR2^b^ = 0.0835 | R1^a^ = 0.0525, wR2^b^ = 0.1145 |
| R indices (all data) | R1 = 0.0611, wR2 = 0.1402 | R1^a^ = 0.0331, wR2^b^ = 0.0839 | R1^a^ = 0.0891, wR2^b^ = 0.1301 |
| Extinction coefficient | n/a | 0.0034(4) | n/a |
| Largest diff. peak and hole [e∙Å^-3^] | 0.263 and -0.294 | 0.273 and -0.154 | 0.359 and -0.230 |

^a^ R1 =Σ⎪⎢*F_0_*⎢– ⎢*F_c_*⎢⎢/Σ⎢*F_0_* ⎢ ^b^ wR2 = [Σw(*F_0_*^2^ – *F_c_*^2^)^2^/Σ(w(*F_0_*^2^)^2^)]^1/2^
